# Reduction of the HIV-1 reservoir in T cells from people with HIV-1 on suppressive antiretroviral therapy using expanded natural killer cells

**DOI:** 10.1128/mbio.02956-25

**Published:** 2026-02-05

**Authors:** Mary Ann Checkley, Benjamin G. Luttge, Cheryl M. Cameron, Konstantin S. Leskov, Curtis Dobrowolski, David N. Wald, Deborah McMahon, Ghady Haidar, Michele D. Sobolewski, P. Nathan Enick, Joshua Cyktor, John W. Mellors, Jonathan Karn

**Affiliations:** 1 Department of Molecular Biology and Microbiology, Case Western Reserve University School of Medicine 196211, Cleveland, Ohio, USA; 2 Department of Nutrition, Case Western Reserve University School of Medicine 682973, Cleveland, Ohio, USA; 3 Department of Pathology, Case Western Reserve University School of Medicine 242911, Cleveland, Ohio, USA; 4 Department of Pathology, Louis Stokes Cleveland VA Medical Center 20083, Cleveland, Ohio, USA; 5 Division of Infectious Diseases, Department of Medicine, University of Pittsburgh School of Medicine 271847, Pittsburgh, Pennsylvania, USA; Tsinghua University, Beijing, China

**Keywords:** human immunodeficiency virus, latency-reversing agents, NK cells, Intact proviral assay, EDITS assay, ADCC, broadly neutralizing antibodies, shock and kill strategy

## Abstract

Treatment with latency-reversing agents (LRAs) alone has been ineffective in reducing HIV-1 reservoirs in people living with HIV-1 (PLWH) who are on antiretroviral therapy (ART), due to inefficiencies in reservoir reactivation and adaptive immune responses. However, NK cells activated with cytokines may be able to target HIV-1 reservoirs more effectively. To explore the therapeutic potential of NK cells, we expanded blood NK cells from multiple donors *ex vivo* into CD56^bright^ CD16^+^ “eNK” cells using artificial antigen-presenting cells (aAPCs) expressing membrane-bound IL21. eNK cells express multiple activating receptors and are highly cytotoxic against specific target cells. They can also kill HIV-infected CD4+ T cells via antibody-dependent cell-mediated cytotoxicity (ADCC) using broadly neutralizing antibodies (bNAbs) against HIV-1 Env gp120/gp41. Notably, eNK cells from PLWH on ART efficiently killed autologous HIV-1+ T cells reactivated by a combination of vorinostat (SAHA) and IL-15 or an IL-15 superagonist (N-803), as evidenced by declines in proviral load, inducible HIV-1 mRNA, and virus release. Adoptive immunotherapy with eNK cells combined with LRA treatment thus presents a promising strategy to reduce the latent HIV-1 reservoir in PLWH.

**IMPORTANCE:**

Antiretroviral therapy (ART) lowers HIV levels in the blood to nearly undetectable amounts, but stopping therapy almost always leads to HIV rebounding in the bloodstream. DNA and RNA tests show that most people living with HIV (PLWH) on ART retain long-lasting HIV reservoirs that remain hidden from the immune system when no HIV is being produced. Eradicating HIV might look like “drug-free remission,” where HIV reservoirs are kept under control by the immune system even if ART is stopped indefinitely. Current strategies for this potential eradication include using HIV latency-reversing agents (LRAs), *ex vivo* expansion of natural killer (NK) cells, and improving the ability to kill infected cells with broadly neutralizing antibodies against HIV. Here, we demonstrate that NK cells from PLWH can be expanded outside the body into “eNK” cells that specifically attack HIV-infected cells without harming uninfected ones, significantly reducing HIV reservoirs *in vitro* after LRA treatment.

## INTRODUCTION

Despite the success of antiretroviral therapy (ART), HIV reservoirs that evade immune detection persist (1). HIV eradication strategies using latency-reversing agents (LRAs) (2, 3) as monotherapy have failed to reduce HIV reservoirs (4–13), due to poor immune cell activation, cytotoxic T lymphocyte (CTL) escape mutants (14), and weak CTL activity (15). Therefore, an effective HIV eradication might require combining LRA therapy with improved immune cell effector function.

Peripheral blood NK cells (CD3^−^ CD56^+^) are ~90% CD56^dim^ and ~10% CD56^bright^ (16). CD56^dim^ NK cells induce apoptosis in target cells using cytotoxic granules or death-receptor ligands (17) and can mediate antibody-dependent cell-mediated cytotoxicity (ADCC) via engagement of the Fcγ-III receptor (CD16). Many broadly neutralizing antibodies (bNAbs) from PLWH have already been shown to induce ADCC, suppress viremia, block infection, and delay viral rebound during analytical treatment interruption (18–34). Although the CD56^bright^ subset of NK cells is more immunoregulatory, some cytokines can induce cytotoxicity (35–38). Since NK cells use activating and inhibitory receptors to identify target cells rather than relying on antigen recognition, they can also target CTL-escape mutants (39). NK cell-mediated cytotoxicity occurs only when more activating receptor ligands are present on target cells than inhibitory receptor ligands. The expression of specific HIV-1 proteins is enough to trigger NK cell recognition through this mechanism. HIV-1 Nef and Vpu can stimulate NK cell recognition by downregulating MHC-I, which is an inhibitory receptor ligand for NK cells (40–43). HIV Vpr and cell stress also upregulate ligands for NK cell-activating receptors (44).

Clinically, adoptive transfer of eNK cells is already used in cancer therapy and could potentially be adapted to target HIV reservoirs *in vivo*. Therefore, we are testing whether eNK cells from PLWH can reduce HIV reservoirs *in vitro*. In PLWH, dysfunctional CD56-negative NK cells accumulate while cytotoxic NK cells decrease (45–47). NK cells from PBMCs have relatively low cytotoxicity in the absence of activation (48) but can be activated *in vitro* by IL-2, IL-15, or IL-15 derivatives (N-803) (49–53). Cytotoxic NK cells isolated directly from PBMCs would not be sufficient to deliver a therapeutic dose without significant activation and expansion. We used artificial antigen-presenting cells (aAPCs) that express membrane-bound IL-21 (mbIL21) to expand NK cells from PBMCs of PLWH or HIV-negative donors and analyzed inhibitory and activating receptor expression levels, cytotoxicity, and ADCC with anti-HIV bNAbs (48, 54, 55). These autologous eNK cells significantly reduced latent HIV reservoirs *in vitro* after proviral reactivation. Similar results were obtained using Good Manufacturing Practice (GMP) and Food and Drug Administration (FDA)-approved NKF cells to produce eNK cells (56), suggesting that the adoptive transfer of eNK cells could become a viable component of HIV eradication.

## RESULTS

### Expansion and activation of NK cells from PLWH using mbIL21 aAPCs yields a highly activated phenotype

**Fig 1:**
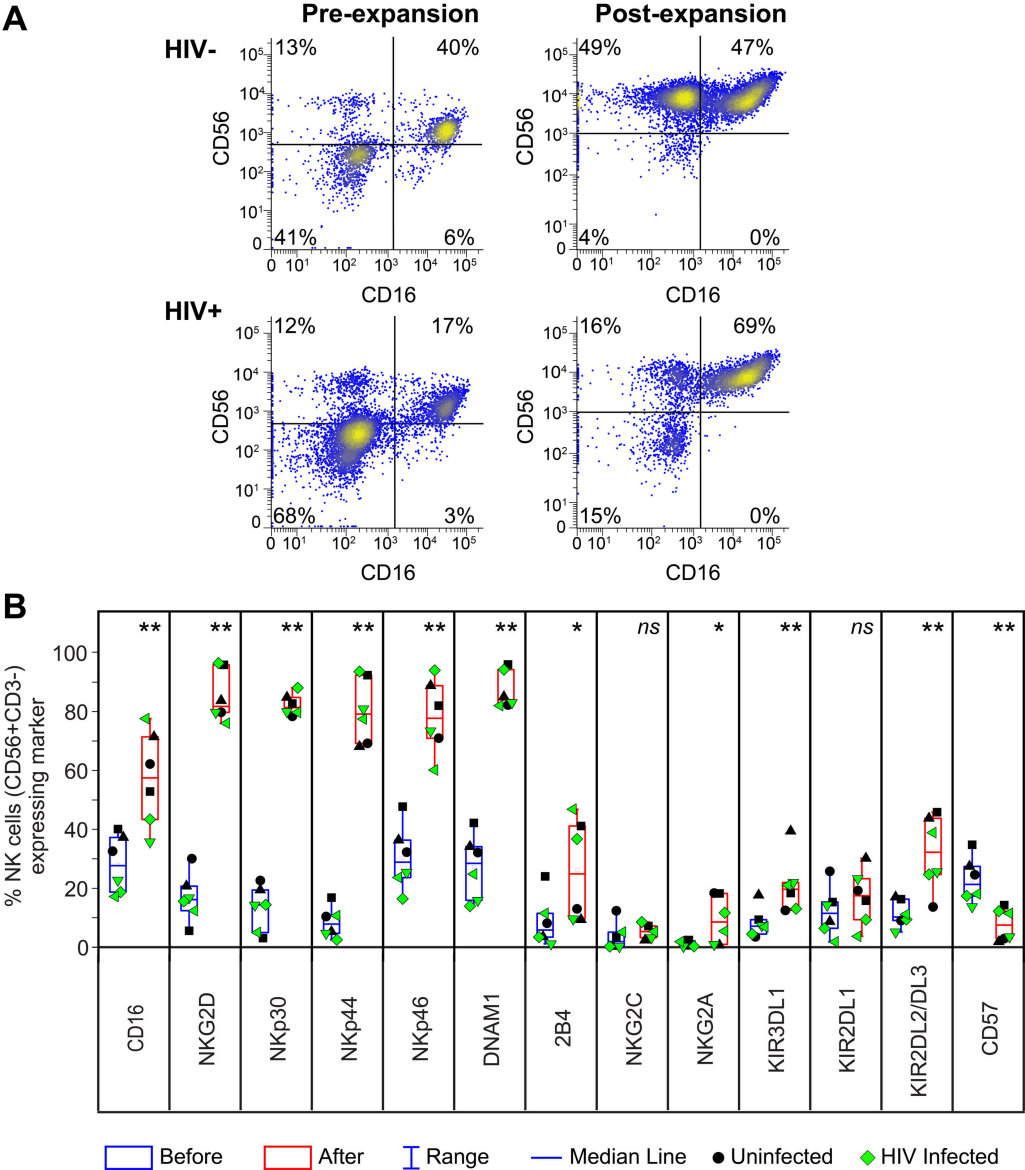
Expansion of NK cells from PLWH yields NK cells that express high levels of activating receptors. (**A and B**) NK cell phenotypic analysis by flow cytometry before and after expansion with irradiated C9.K562.mbIL21 cells. (**A**) Representative dot plots gated on CD3^−^ cells showing expression of NK cell markers CD56 and CD16 before and after (pre and post) expansion from representative HIV− donor and PLWH. (**B**) Frequency of CD56^+^ NK cells expressing NK cell markers before (blue box) and after expansion (red box) from HIV− donor (black symbols) and PLWH (green symbols). In total, *n* = 3 biological replicates per group. Significant differences after expansion were determined using paired *t*-tests, *P < 0.05, **P < 0.01, and ns, non-significant.

We explored whether cytotoxic NK cells from PLWH could be expanded and activated *ex vivo* for adoptive immunotherapy. We isolated NK cells from PBMCs of well-suppressed ART-treated PLWH or HIV-negative controls and cultured them with K562 feeder cells expressing membrane-bound IL21 (C9.K562-mbIL21) for 3–4 weeks, leading to a 150-fold to 500-fold expansion of eNK cells that were analyzed for the expression of CD56, CD16, and other NK cell receptors (Fig. 1A and B). Chronic HIV infection reduces cytotoxic NK cell subsets (CD56^dim^ CD16^+^) (57). Our freshly isolated NK cells were primarily CD56^dim^ CD16^+^ or CD56^−^CD16^−^, with the CD56-negative subset being more prevalent in PLWH but becoming mostly CD56^bright^ CD16^+^ (76%–98%) after expansion, regardless of HIV status.

CD56^+^ eNK cells expressing NK cell-activating receptors were significantly more abundant after expansion, including CD16 (2-fold), NKG2D (7-fold), NKp30 (11-fold), NKp44 (15-fold), NKp46 (3-fold), DNAM1 (4-fold), and 2B4 (5-fold) (Fig. 1B), along with higher expression levels/cell (Fig. S1A). The proportion of CD56^+^ eNK cells expressing inhibitory receptors also modestly increased, including KIR3DL1 (3-fold), KIR2DL2/DL3 (3-fold), and NKG2A (8-fold). The eNK cells were mostly negative for CD57, a marker of NK cell maturation associated with reduced proliferation and cytokine responsiveness (58). We also examined eNK cells expanded with an alternative aAPC cell line expressing mbIL-21 (NKF cells) that has been certified as an investigational new drug (IND) (56). This yielded eNK cells with a very similar phenotype (Fig. S1B). In summary, expanding NK cells from PLWH consistently produced CD56^bright^ CD16^+^ eNK cells with high levels of activating receptors while preserving inhibitory receptors important for regulation.

### eNK cells are highly cytotoxic and compatible with LRAs

**Fig 2:**
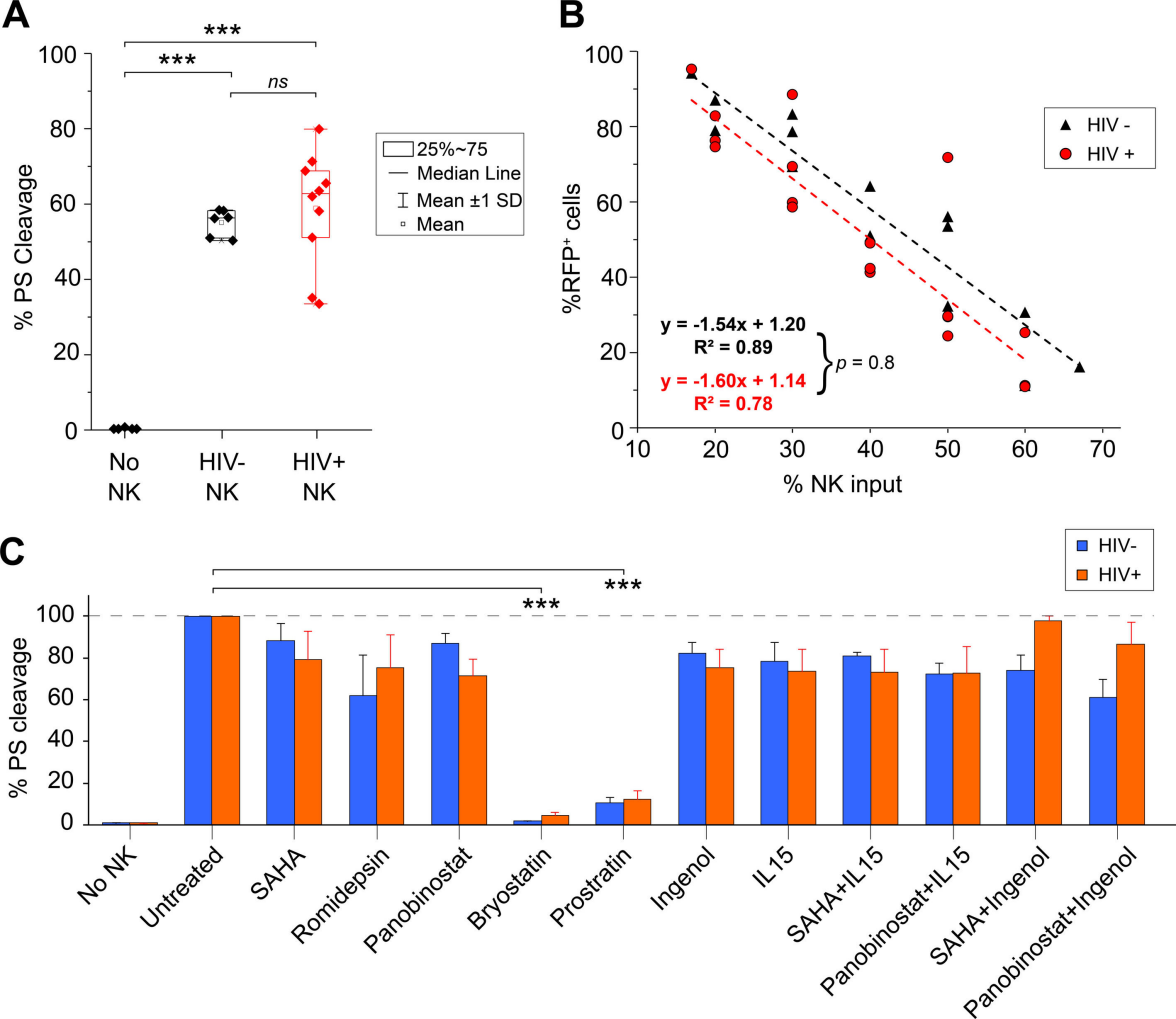
eNK cells from PLWH are highly cytotoxic against K562 target cells and their cytotoxicity is not affected by most LRAs. (**A–C**) eNK cells from PLWH and HIV− donors were cocultured with K562 cells and killing was measured by flow cytometry. (**A**) Killing of K562 target cells by eNK cells at a 2:1 (E:T) ratio is measured by PanToxiLux substrate (PS) cleavage. The percentage of K562 cells with PS cleavage is shown. In total, *n* = 5 biological replicates for HIV-negative group and *n =* 10 biological replicates for HIV-positive group. Each sample was performed in triplicate. (**B**) CTV-stained eNK cells were cocultured with K562-RFP target cells. Cytotoxicity was then detected in target cells (CTV− events) by loss of RFP; *n* = 3 biological replicates per group. Each sample was performed in triplicate. There was no significant difference in cytotoxicity between eNK cells from PLWH or HIV− donors (*P* = 0.8). (**C**) Effect of LRAs on eNK cell cytotoxicity. eNK cells from PLWH or HIV− donors were treated with LRAs for 24 h or left untreated, then washed and cocultured with K562 cells at a 2:1 (E:T) ratio to measure cytotoxicity by PS cleavage. LRAs were used alone or in combination as follows: SAHA (335 nM), romidepsin (10 nM), panobinostat (20 nM), bryostatin (5 nM), prostratin (1 µM), ingenol (100 nM), and IL15 (10 ng/mL). Percentage of K562 cells targeted for killing (% PS cleavage) by eNK cells relative to no LRA treatment (untreated) is shown.In total, *n* = 2 biological replicates from three independent experiments for HIV-negative group and *n* = 3 biological replicates for HIV-positive group. Error bars show SEM. Significant differences were determined using two-tailed unpaired *t*-tests of the means in panel A, or the slopes created by linear regression in panel B, and paired *t*-tests in panel C, *** *P* < 0.001.

Next, we assessed the cytotoxicity of eNK cells from PLWH against target cell lines. Using a PanToxiLux substrate (PS) to detect granzyme B delivered into target cells, eNK cells from PLWH killed 55%–59% of K562 cells at a 2:1 (eNK:K562) ratio based on PS cleavage (Fig. 2A; Fig. S2A and B). We also measured cytotoxicity against our novel K562-RFP cell line, where the loss of RFP directly correlates with the gain of a dead cell stain (FVD) (Fig. 2B; Fig. S2) and found that 40%–45% eNK cell input was required to kill 50% of target cells. With either method, eNK cells from PLWH were just as cytotoxic as those from HIV-negative donors, indicating that NK cells from PLWH can be effectively expanded into highly cytotoxic eNK cells.

To visualize eNK cell cytotoxicity via time-lapse microscopy, TNFα-activated 3C9 cells were combined with a dead-cell stain (Sytox Orange) and imaged at 37°C, either alone or with CTV-labeled eNK cells. Without eNK cells, 3C9 cells remained uniformly distributed and viable (Video S1). In contrast, eNK cells were highly motile and conjugated target cells within 10 min (Fig. S2D). At a 1:4 (NK:3C9 cell) ratio, 6%–12% of 3C9 cells were killed within 45 min (Fig. S2D, Video S2). Some eNK cells killed up to four target cells during this period, demonstrating that eNK cells are highly motile and capable of serial killing.

Since NK cells would be exposed to LRAs during a shock and kill approach, we evaluated the effects of clinically relevant concentrations of HDACi (SAHA, panobinostat, and romidepsin) and protein kinase C agonists (bryostatin, prostratin, and ingenol) on eNK cell-mediated cytotoxicity (Fig. 2C). IL-15 activates both NK and T cells and enhances latency reversal; hence, we tested the cytotoxicity of eNK cells after IL-15 treatment alone or with SAHA or panobinostat. HDACi or ingenol treatment of eNK cells resulted in only a modest decrease in cytotoxicity, which was not affected by IL-15. Combining ingenol with HDACi was most compatible with eNK cells from PLWH, whereas bryostatin or prostratin dramatically reduced cytotoxicity. Therefore, any HIV reactivation strategy using LRAs should consider the impact on NK cell effector functions.

### eNK cells express markers of cytotoxicity, glycolysis, oxidative phosphorylation, and cell proliferation

**Fig 3:**
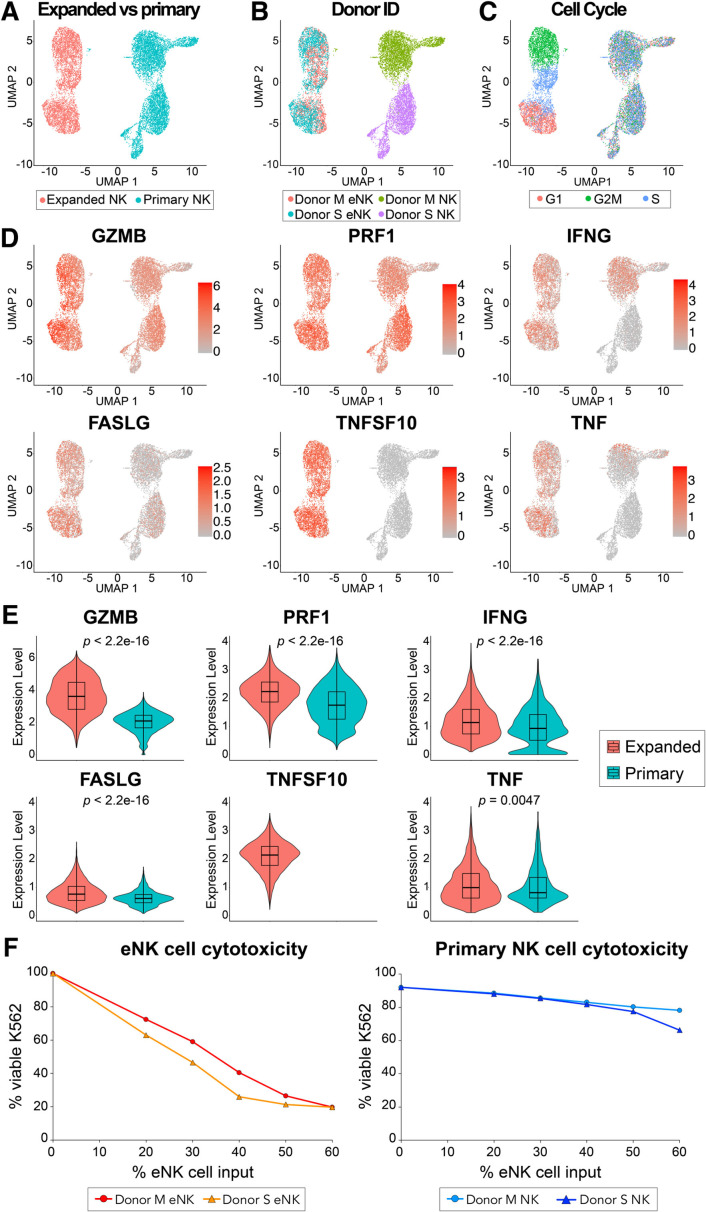
scRNA-seq reveals upregulation of markers involved in cytotoxicity in eNK cells. NK cells from 2 PLWH were analyzed by 10× Genomics scRNA-seq before (primary NK) and after expansion (eNK). (**A**) UMAP of all events of 12,873 NK cells (primary shown in blue and expanded shown in red). (**B**) UMAP with events color coded to indicate donor origin before (NK) and after expansion (eNK) of NK cells. (**C**) UMAP with events color coded by cell cycle phase. (**D**) Feature plots showing the expression levels of selected genes whose functions are crucial in NK cell-mediated cytotoxicity for both eNK and primary NK cells: granzyme B (GZMB), perforin (PRF1), IFN-γ (IFNG), Fas ligand (FASLG), TRAIL (TNFSF10), and TNF-α (TNF). (**E**) Violin plots of selected markers involved in cytotoxicity. *P*-values were calculated by the Wilcoxon rank-sum test using Seurat package for R. Raw counts of each transcript in single cells were subjected to Bayesian denoising using DecontX package for R. (**F**) NK cell-mediated cytotoxicity against K562. A fraction of NK cells before expansion (primary NK cells, graph on the right) and after expansion (eNK cells, graph on the left) that were analyzed by scRNAseq was also analyzed in parallel for cytotoxicity. NK cells were stained with CTV and cocultured with K562-RFP target cells. Cytotoxicity in target cells (CTV− events) was detected by loss of RFP. Each sample was analyzed in triplicate. *n* = 2 biological replicates from PLWH.

Single-cell RNA sequencing (scRNA-seq) revealed that eNK cells have a distinct transcriptional profile compared with non-expanded primary NK cells from PLWH (Fig. 3A). Unlike primary NK cells, eNK cells did not segregate based on Donor ID (Fig. 3B) but clustered based on cell cycle markers using Seurat analysis (Fig. 3C). NK cells kill target cells by releasing cytolytic granules, engaging death receptors, or releasing cytokines to promote cytotoxicity. When analyzed for these markers of cytotoxicity, eNK cells expressed significantly higher levels of granzyme B (GZMB), perforin 1 (PRF1), Fas ligand (FASLG), TRAIL (TNFSF10) (Fig. 3D and E), gamma-interferon (IFNG and IFNγ), and TNFα (TNF). Using a 50% NK cell input in our K562-RFP assay (Fig. 3F), eNK cells from DonorID Donor M or Donor S killed 80% of target cells, compared with 20% killing with primary NK cells. In primary NK cells (pre-expansion), there was donor-specific variability in expression of cytotoxic markers but no significant difference in actual cytotoxicity.

**Fig 4:**
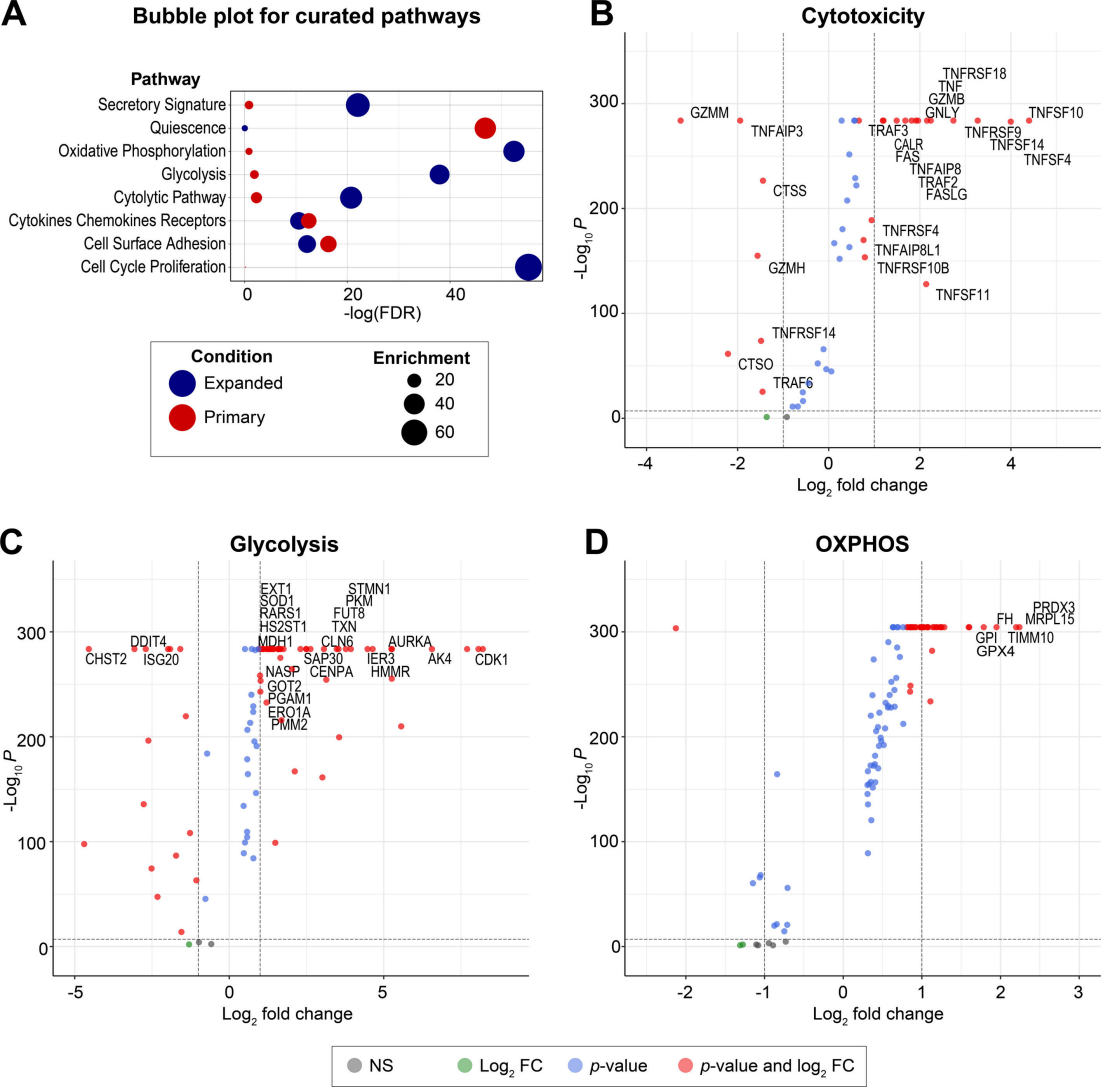
eNK cells express markers that mediate cytotoxicity and metabolism. (**A**) Bubble plot displaying pathway enrichment analysis using curated pathways of primary NK cells before expansion (red) and after expansion (blue). The size of each bubble corresponds to the degree of enrichment for each pathway, that is, the percentage of overexpressed marker genes from a particular pathway in a sample determined using the Seurat package for R relative to the total number of genes in the same curated reference pathway. Negative log of False Discovery Rate (FDR) statistics was calculated for each pathway using the hypeR package for R. (**B–D**) Volcano plots displaying differentially expressed (DE) genes in eNK cells compared to primary NK cells, based on curated pathways of (**B**) cytotoxicity, (**C**) glycolysis, and (**D**) oxidative phosphorylation (OXPHOS). Each colored dot represents a DE gene. Genes with >1 log_2_ fold change (FC) in expression and a significant *P*-value (*P* < 1 × 10^−7^, Wilcoxon rank-sum test) are in red. Genes with a significant *P*-value but <1 log_2_ FC are in blue. Genes with > 1 log_2_ FC but a non-significant *P*-value are in green. Genes with both a non-significant *P*-value (NS) and <1 log_2_ FC are in gray. Names of the most pertinent markers are displayed. Due to analytical limitations, genes with *P* < 1 × 10^−300^ are aggregated at a maximum of *P* = 1 × 10^−300^.

scRNA-seq identified 3851 upregulated and 1,995 downregulated genes in eNK cells (log_2_ fold change > 0.5 and Bonferroni-adjusted *P* < 0.05, Wilcoxon rank-sum test) (Fig. S3A). Using NK cell-specific curated pathways (59) (Fig. 4A), we found that secretory signature, oxidative phosphorylation (OXPHOS), glycolysis, cytolytic, and cell cycle proliferation pathways were significantly enriched in eNK cells. In contrast, primary NK cells were enriched in the quiescence pathway. In addition to the markers already discussed (Fig. 3), eNK cells also expressed several other markers in the cytotoxicity pathway (Fig. 4B), including calreticulin (CALR), FAS, granulysin (GNLY), TNF-superfamily (TNFSF4, 10, 11, and 14), TNFα-induced protein-18 and its ligand (TNFAIP8 and TNFAIP8L1), TNF-receptor superfamily (TNFRSF4, 9, 10B, and 18), and TNF-receptor-associated factors (TRAF2 and 3).

Glycolysis and OXPHOS pathways were also enriched in eNK cells (Fig. 4C and D). Many upregulated genes in these pathways are also linked to cell division (e.g., cyclin-dependent kinase 1 [CDK1], Aurora A kinase [AURKA]), consistent with the mitogenic effects of C9.K562.mbIL21 aAPCs. However, activation also boosts mitochondrial metabolism and glycolysis (60, 61). While CDK1 is primarily known for its role in regulating cell proliferation (62), it also controls glycolysis by phosphorylating FBA1, PGK1, and GPH1 (63, 64). AURKA is associated with both cell cycle progression (65) and glycolysis (66–68). Upregulation of FUT8 in eNK cells is notable, since fucosylation promotes NK cell cytotoxicity (69). Thioredoxin (TXN) upregulation is consistent with activation and resists oxidative stress (70). Secretory pathways, cell cycling, cell adhesion, cytokines/chemokines and their receptors were all generally upregulated in eNK cells, and quiescence was downregulated (Fig. S3B through F). CCL3 (MIP-1α), which increases cytotoxicity and blocks HIV spreading in target cells *in vitro* (71, 72), was upregulated in eNK cells (S3D Fig). Several genes upregulated in the NK-specific curated pathways (Fig. 4; Fig. S3) were also enriched in the Hallmark gene sets from the Human Molecular Signatures Database (MSigDB) (Fig. S4), including DNA repair, E2F targets, G2M-checkpoint, IL2-STAT5 signaling, MTORC1-signaling, MYC targets v1/v2, OXPHOS, and unfolded protein responses (Fig. S4 and S5). Unfortunately, when using Hallmark gene sets, it is unclear which genes are most relevant to cytotoxicity because of gene overlap across multiple pathways. However, the NK cell-specific curated pathway analysis more clearly showed that eNK cells from PLWH upregulate key markers of cytotoxicity, metabolism, and cell proliferation.

### eNK cells specifically kill acutely HIV-1-infected T cells

**Fig 5:**
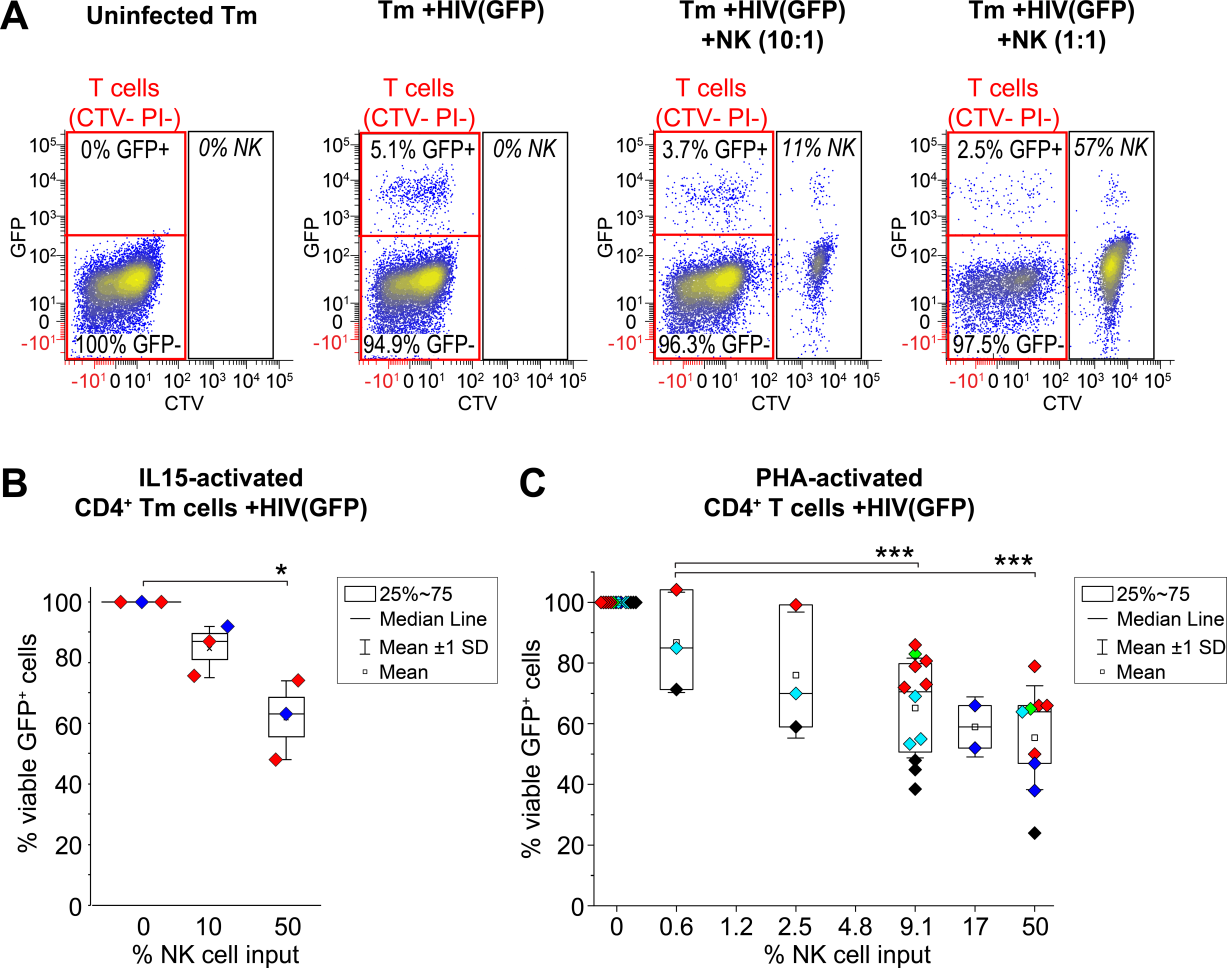
eNK cells specifically and efficiently kill autologous CD4+ T cells acutely infected with HIV-1. (**A–C**) CTV-stained eNK cells were cocultured with autologous primary T cells infected with replication-competent HIV-1 expressing GFP [HIV(GFP)]. Using flow cytometry, killing of HIV-infected T cells was detected by loss of GFP and total target cell death was detected by PI staining of CTV− cells. (**A**) Representative dot plots of uninfected memory T cells (Tm), HIV(GFP)-infected Tm cells [Tm +HIV(GFP)] alone or after 1-day coculture with eNK cells at 1:10 and 1:1 (NK:Tm) ratios. Percentages of GFP+ and GFP− Tm cells are highlighted by red gates. CTV+ eNK cells are shown but excluded from analysis. (**B and C**) Box plots showing dose-dependent, eNK cell-mediated killing of activated CD4+ T cells infected with HIV(GFP). The percentage of viable GFP+ Tm cells (CTV− PI−) in cultures with eNK cells was normalized to cultures without eNK cells. (**B**) eNK cell-mediated killing of IL15-activated memory T cells (Tm) infected with HIV(GFP); *n* = 3 independent experiments, and each biological replicate (shown in unique colors) was performed in triplicate. (**C**) eNK cell-mediated killing of PHA-activated CD4+ T cells infected with HIV(GFP). In total, *n* = 2–12 independent experiments for each dose of NK cells (symbols show mean of each sample performed in triplicate) using five biological replicates (unique color for each). Significant differences were determined using paired *t*-tests, *P < 0.05, ***P < 0.001.

To determine whether eNK cells specifically kill HIV-1-infected cells instead of uninfected cells, we treated CD4+ memory T (Tm) cells with IL-15 and infected them with R5-tropic HIV-GFP (Fig. 5A). HIV-infected Tm cells (1%–5% GFP+) were cocultured with CTV-stained eNK cells for 24 h, and specific killing of HIV+ cells was detected by GFP loss. Staining with propidium iodide (PI) indicated cytotoxicity against all cells. We consistently observed 38% killing of HIV+GFP+ Tm cells with a 50% NK cell input (1:1 E:T ratio), and 15% killing with a 10% NK cell input (Fig. 5B). PI staining of GFP-negative cells was minimal (Fig. S6A), suggesting highly selective killing of HIV+ cells. To compare with maximal target cell activation, we cultured PHA-activated, HIV-GFP-infected CD4+ T cells (5%–15% GFP+) with autologous eNK cells for 24 h, yielding 45% killing at a 50% NK cell input and 30% killing at a 9.1% NK cell input (Fig. 5C).

Specificity was also demonstrated using a modified PanToxiLux assay. Specific killing of HIV+GFP+ cells correlated with GFP loss, while overall cytotoxicity was indicated by PS cleavage. (Fig. S6B). PHA-activated CD4+ T cells were either acutely infected with X4-tropic or R5-tropic HIV-GFP or transduced with an HIV-1 construct (PHR’) that expresses mCD8α-GFP on the cell surface (Fig. S6C); 5%–7% GFP+ T cells were cocultured overnight with autologous eNK cells, resulting in significant killing of GFP+ CD4+ T cells (~42% of PHR’-transduced cells and ~54% of HIV-GFP-infected cells) (Fig. S6D), compared to <1% killing of uninfected (GFP-negative) cells detected by PS cleavage (Fig. S6C). NKF-expanded NK cells also specifically targeted PHA-activated, autologous HIV(R5)-GFP-infected CD4+ T cells for killing (Fig. S6E). Overall, these data indicate that eNK cells can specifically target and eliminate HIV+ cells without harming uninfected cells.

### ADCC enhances eNK cell-mediated killing of HIV-infected cells

**Fig 6:**
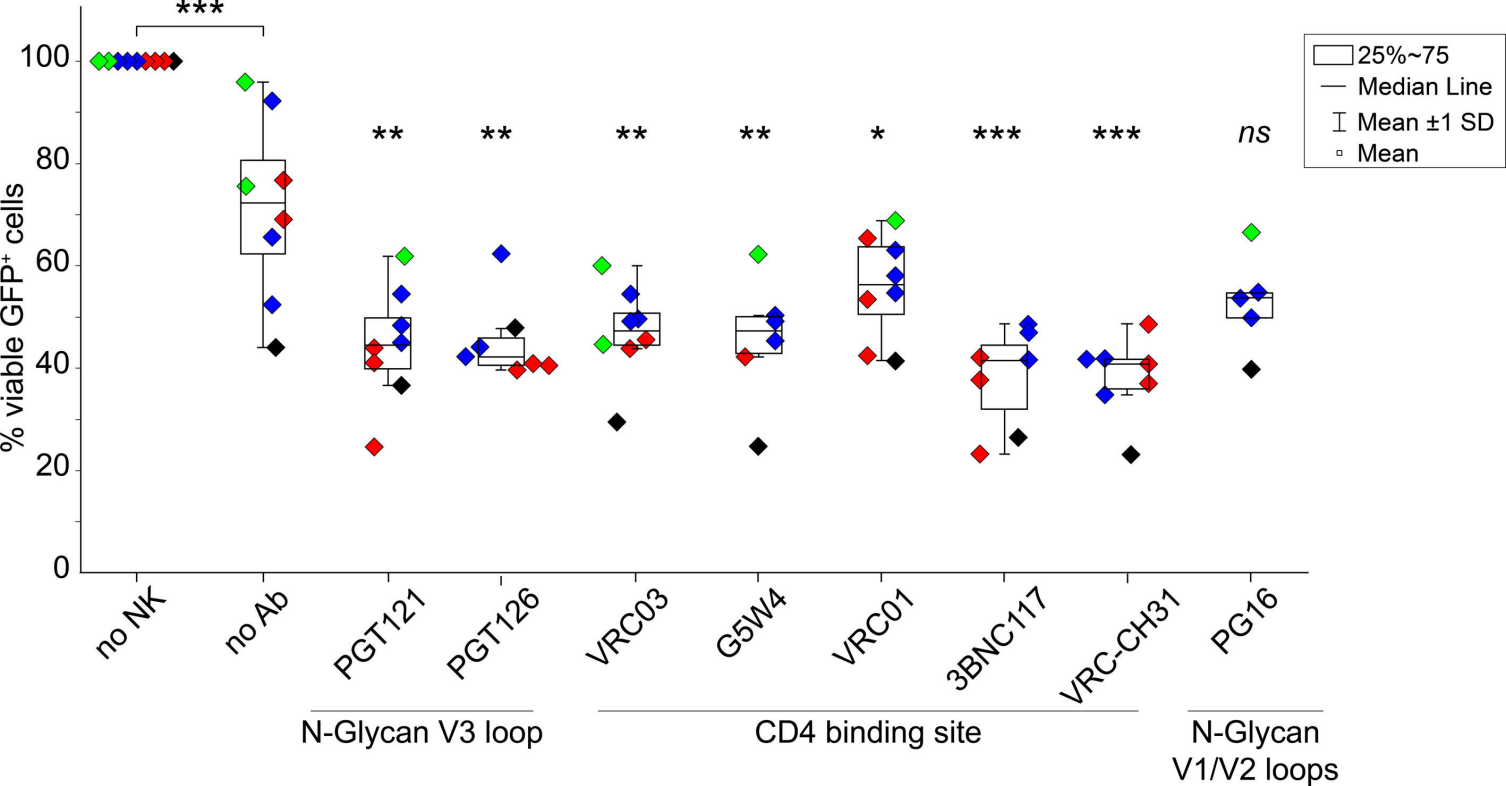
eNK cell-mediated killing of HIV-infected cells can be enhanced with ADCC using bNAbs against HIV Env. PHA-activated CD4+ T cells were acutely infected with replication-competent R5-tropic HIV(GFP) and cultured for 1 day with CTV-stained eNK cells at a 1:10 (NK:T) ratio mixed with anti-HIV Env at 10 µg/mL and 10 µM raltegravir. By flow cytometry, killing of HIV-infected T cells (CTV−) is detected by loss of GFP, and total target cell death is detected by PI staining. The percentage of viable GFP+ T cells (CTV− PI−) in cultures with eNK cells was normalized to cultures without eNK cells; *n* = 4 biological replicates. Each donor is shown in a different color, and each symbol represents the mean of samples in triplicate from each independent experiment. Significant differences were determined using unpaired *t*-tests, * *P* < 0.05, ** *P* < 0.01, *** *P* < 0.001, *ns*, non-significant.

To determine whether eNK cells kill HIV-infected cells through ADCC, we selected anti-HIV Env bNAbs targeting the V3 loop (PGT121, PGT126, PGT128, PGT135, 10-1074, and 2191), CD4 binding site (VRC01, G54W, VRC03, CH106, 3BNC117, and VRC-CH31), V1/V2 loops (PG9, PG16, PG145, HG107, and CH58), and gp41/MPER (7B2, 7B2-AAA, 2F5, 7H6, and 10E8). PHA-activated CD4+ T cells acutely infected with X4-tropic or R5-tropic HIV-GFP were mixed with bNAbs and autologous eNK cells at ratios of 1:10 (E:T). bNAbs were deemed ADCC-compatible only if they increased killing compared to negative controls (human anti-H1N1 influenza A or no antibody). CD4 binding site bNAbs induced ADCC equally against HIV (R5) and (X4)-infected cells (Fig. S7A). V3 loop bNAbs were only effective against HIV(R5)-infected cells, as expected. Some V1/V2 loop (PG16) and MPER (2F5 and 10E8) bNAbs also elicited ADCC against both HIV(R5) and (X4)-infected cells. Negative controls elicited no ADCC (Fig. S7B). ADCC was consistent with eNK cells from multiple donors. Although some NK cell-mediated killing occurred at a low effector:target ratio (1:10) without antibody, each bNAb tested (except PG16) showed statistically significant enhancement of target cell killing (Fig. 6). We titrated selected bNAbs (0.16–10 μg/mL) and found ADCC over a wide range using a 1:10 (E:T) ratio, with saturation at 1–2.5 μg/mL. VRC01 was most potent, with maximal ADCC at 0.625 μg/mL (Fig. S7C). Thus, anti-HIV Env bNAbs enhanced killing of HIV-infected CD4+ T cells by eNK cells.

### Autologous eNK cells from PLWH reduce HIV release from CD4+ T cells after latency reversal

**Fig 7:**
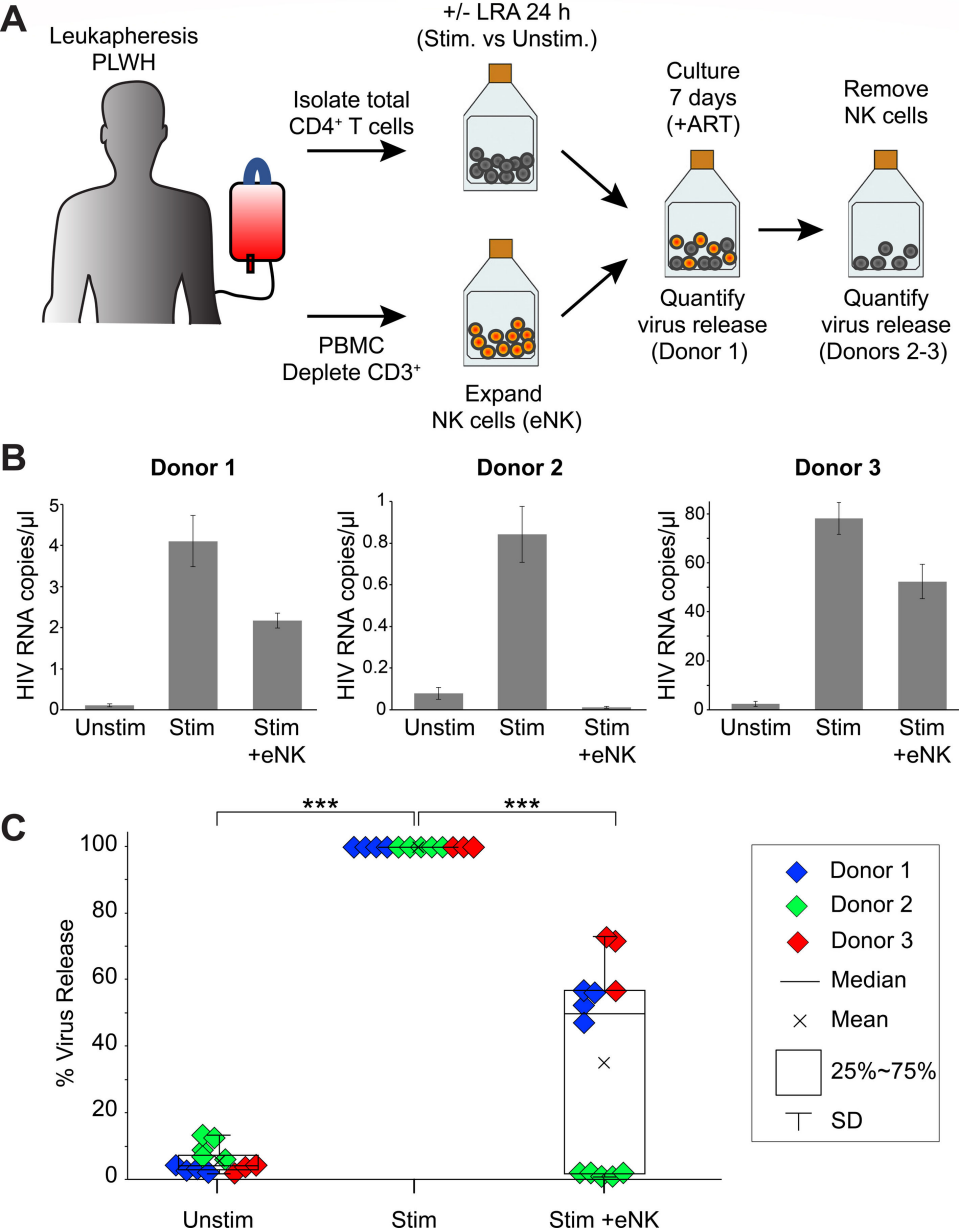
eNK cells from PLWH reduce HIV release from autologous CD4+ T cells after latency reversal. (**A**) Schema of eNK killing assays detected by virus release. CD4+ T cells from ART-treated PLWH were isolated from PBMCs and activated with LRA for 24 h. In parallel, NK cells were enriched from PBMCs by CD3 depletion and expanded into eNK cells using NKF cells. Cocultures of activated CD4+ T cells were mixed with an equal number of autologous eNK cells and incubated for 7 days in growth media containing antiretroviral drugs. After the coculture, supernatants were collected to measure virus release (Donor 1). For Donors 2 and 3, eNK cells were removed at day 7 and incubated for an additional 7 days prior to collection of supernatants. (**B and C**) Virus release measurements in CD4+ T cells from PLWH. Supernatants from CD4+ T cells cultures alone or with eNK cells were collected to quantify HIV release by detecting HIV RNA in the supernatant using the Aptima HIV-1 Quant assay. After complete media change at day 5 (Donor 1) and day 8 (Donors 2–3), *de novo* virus release was detected at day 7 (Donor 1) and day 14 (Donors 2–3). (**B**) Virus release in CD4+ T cells from PLWH after eNK cell treatment. Bar graphs show HIV RNA copies/μL for unstimulated CD4+ T cells (Unstim) compared to stimulated CD4+ T cells alone (Stim) or with eNK cells (Stim+eNK) from each donor. (**C**) Percent virus release was normalized to stimulated CD4+ T cells (Stim) and compared to stimulated CD4+ T cells with eNK cells (Stim+eNK). CD4+ T cells with no stimulus (Unstim) were also tested as a control for HIV latency in PLWH samples; *n* = 3 biological replicates. Significant differences were determined using paired *t*-tests. *** *P* < 0.001. Donor 1 *n* = 4 replicates per sample, Donor 2 *n* = 9 replicates per sample, and Donor 3 *n* = 3 replicates per sample. Error bars represent STDEV.

As a preclinical model for HIV eradication, we combined GMP for NK cell expansion, clinically used LRAs, and a clinical assay for HIV detection. NK cells were expanded from PBMCs of PLWH (Donors 1, 2, and 3) using FDA-approved NKF cells (Fig. 7A), yielding ~35-fold expansion of CD3-negative CD56^bright^ CD16^+^ eNK cells with <0.1% T cell contamination (Fig. S8A). GMP-eNK cells were similar to eNK cells expanded with the C9.K562-mbIL21 aAPC (compare Fig. 1A) and efficiently killed K562-RFP cells (Fig. S8B). 35%–38.5% GMP-eNK cell input was required to kill 50% of K562-RFP target cells, comparable to NK cells expanded with C9.K562-mbIL21 aAPC (compare Fig. 2B).

To demonstrate the antiviral activity of GMP-eNK cells, we measured HIV release after latency reversal (Fig. 7A). Total CD4+ T cells from PLWH Donors 1–3 were activated overnight using TCR stimulus or LRA treatment, then cocultured with autologous eNK cells and antiretroviral drugs. Target cells from Donor 1 were maximally activated by TCR stimulation. For Donors 2 and 3, we combined SAHA with the IL-15 superagonist N-803, which has already demonstrated promising results *in vivo* and *in vitro* by inducing HIV mRNA expression (Fig. S8C) (73–76). HIV RNA release was measured using the Aptima HIV-1 Quant assay after coculture with or without eNK cells. For Donor 1, media was replaced on day 5, and the cultured supernatant was analyzed on day 7. For Donors 2 and 3, eNK cells were removed on day 7 by CD56^+^ depletion (Fig. S8D), and the supernatant from the remaining T cells was analyzed after 7 days, and virus release ranged from 0.84 to 78 HIV RNA copies/µL (Fig. 7B). Data were normalized to stimulated CD4+ T cells without NK cells (Stim) and compared to samples treated with eNK cells (Stim + eNK) (Fig. 7C). Unstimulated samples (Unstim) were cultured in parallel, and HIV release from these cells was minimal, as expected. In contrast, virus release from stimulated cells was highly significant. Compared to samples without NK cell treatment, stimulated CD4+ T cells incubated with eNK cells consistently showed significantly less virus release, with reductions ranging from a modest 1.5-fold to 2-fold (Donors 1 and 3) to nearly complete suppression of virus release (Donor 2). This indicates that eNK cells have notable antiviral activity against reactivated HIV+ CD4+ T cells from PLWH.

### eNK cells from PLWH reduce proviral loads in autologous CD4+ T cells after latency reversal *in vitro*

**Fig 8:**
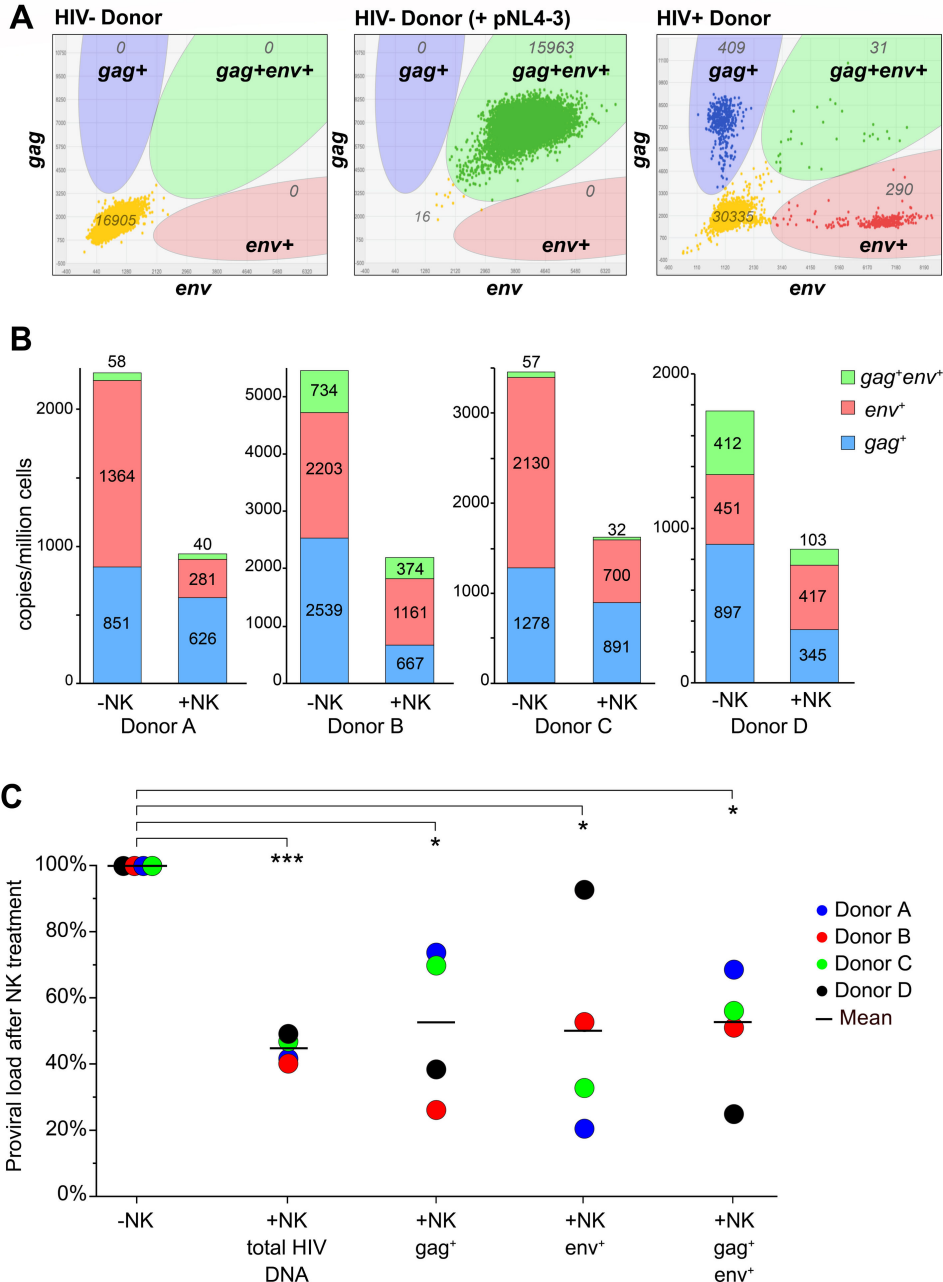
Potentially intact proviral load (PIPL) assay for HIV-1 shows decreases in HIV reservoirs after treatment with eNK cells. (**A**) HIV-1 proviruses in cellular DNA are counted by dPCR using FAM-labeled *gag* and VIC-labeled *env* probes in duplex. PBMC DNA from an HIV-negative donor (left) is a negative control, which when mixed with a trace amount of pNL43 (middle) is a control for fully intact (*gag+env*+) proviruses. Memory CD4+ T (Tm) cell DNA from a PLWH on ART (right) contains an excess of potentially defective *gag+* (*env*-negative) and *env+* (*gag*-negative) HIV proviruses, with a limited number of potentially intact proviruses (*gag+env*+). (**B**) Indirect detection of NK cell-mediated cytotoxicity by the PIPL Assay. Tm cells isolated from PLWH were treated overnight with SAHA+IL15 prior to culture for 7 days without NK cells (−NK) or 1:1 with eNK cells (+NK). After reisolation of Tm cells, cellular DNA samples were analyzed by the PIPL assay, and contributions of *gag+*, *env+*, and *gag+env*+ proviruses to the total are shown. (**C**) Statistical analysis of NK cell-mediated effects on proviral loads. Data from PIPL assays of NK cell-treated Tm cells (% proviral load after NK treatment) were normalized to Tm cells cultured without NK cells (-NK) and averaged. Total HIV DNA is the sum of all proviruses detected. Relative changes in potentially defective (*gag+ or env+*) or potentially intact (*gag+env*+) proviral loads are shown. *n* = 4 biological replicates. Statistical differences in the means of proviral loads +/– NK cells were determined by paired, two-tailed Student’s *t*-tests (*P < 0.05, *** *P* < 0.001).

We developed the potentially intact proviral load (PIPL) assay, which is similar to the intact proviral DNA assay (IPDA) (77, 78), to estimate alteration of HIV reservoirs after NK cell exposure (Fig. 8). Using primer/probe sets targeting both *gag* and *env* in individual proviruses by digital PCR, we detect single-positive *gag+* and *env+* proviruses that are potentially defective, as well as potentially intact double-positive (*gag+env*+) proviruses. To determine whether PCR inhibition at high DNA concentrations could lead to false negatives, we show that the PIPL assay is not restricted by excess uninfected cell DNA at the concentrations used in primary samples. pNL4-3 mixed with HIV-negative DNA in excess yielded only *gag+env*+ signals (>99% PIPL) with no false negatives. In contrast, CD4+ memory T (Tm) cell DNA from well-suppressed PLWH mainly contained single-positive proviruses, and *gag+env*+ proviruses were rare (~4% PIPL) (Fig. 8A).

We isolated Tm cells from PBMCs of four PLWH and treated these with SAHA and IL-15 overnight. Reactivated Tm cells were incubated with eNK cells for 7 days, after which the NK cells were removed (Fig. S8E). DNA from remaining Tm cells was analyzed by the PIPL assay, which detected highly significant losses of total proviral load (56% reduction, *P*<0.001), including potentially defective *gag+* (48% reduction, *P*<0.05) and *env*+ proviruses (50% reduction, *P*<0.05) and potentially intact *gag+env*+ proviruses (50% reduction, *P*<0.05) (Fig. 8B and C). We interpret loss in proviral load as HIV-specific cell death, suggesting that eNK cells can reduce HIV+ reservoirs *in vitro*, including cells with potentially intact proviruses.

### eNK cells from PLWH reduce reservoirs of inducible HIV mRNA in autologous CD4+ T cells after latency reversal

**Fig 9:**
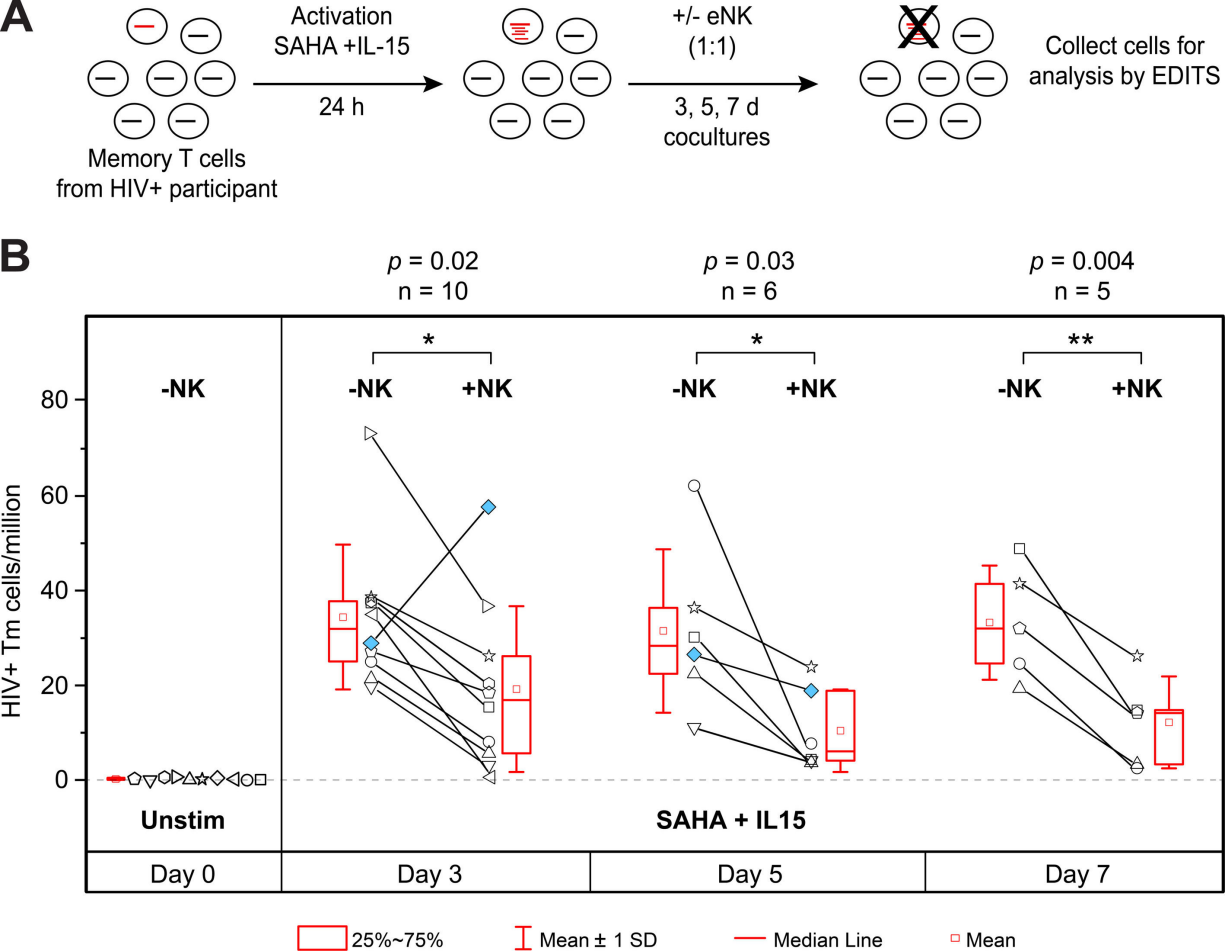
Reduction of latent HIV reservoirs in CD4+ memory T cells from ART-treated PLWH after latency reversal and treatment with autologous eNK cells. (**A**) Schema of the reduction of latent reservoirs in CD4+ memory T (Tm) cells from ART-treated PLWH using eNK cells. Tm cells were treated with SAHA+IL15 for 24 h, then cocultured with autologous eNK cells for 3–7 days. Killing of HIV+ Tm cells was detected by loss of cell-associated singly spliced (SS) HIV *env/vpu* RNA using the EDITS assay. (**B**) Box plot showing loss of SS HIV RNA+ Tm cells after coculture with eNK cells at 3 days (*n* = 10 biological replicates), 5 days (*n* = 6 biological replicates), and 7 days (*n* = 5 biological replicates), measured by EDITS. Each symbol represents a different donor. Statistical differences were determined using paired Student’s *t*-tests, and *P* values are indicated (*P < 0.05, **P < 0.005).

To quantify inducible cell-associated HIV-1 mRNA, we used a next-generation sequencing-based method (EDITS assay) that estimates the number of cells expressing singly spliced (SS) HIV *env* mRNA in latently infected T cells from PLWH in response to HDACi, PKCa, and TCR stimulation (79, 80). The combination of SAHA with IL-15 or N-803 is a particularly potent stimulus, comparable to TCR stimulation (Fig. S8C). We used EDITS to detect SS HIV RNA from memory CD4 T (Tm) cells of PLWH *in vitro* after treating them with SAHA and IL-15, then cultured them alone or with autologous eNK cells in the presence of antiretroviral drugs for 3 days. We observed a 32%-98% reduction in HIV *env* mRNA+ cells (average 44%) in 9 out of 10 PLWH (Fig. 9) relative to Tm cells alone. In a time-course study of Tm cells cultured with eNK cells at an E:T ratio of 1:1, we observed losses of HIV *env* mRNA+ cells at 3 days (56% loss; *P*<0.05), 5 days (67% loss; *P*<0.05), and 7 days (63% loss; *P*<0.005). This indicates significant killing of Tm cells within 3 days that persists for at least 7 days. Even the one sample with higher HIV *env* mRNA+ cells at 3 days eventually began decreasing by day 5. Uninduced cells (unstim) showed virtually no detectable HIV *env* mRNA. Our nucleic acid assays (EDITS, PIPL) demonstrate for the first time that autologous eNK cells can significantly reduce HIV reservoirs in LRA-treated Tm cells *in vitro*.

## DISCUSSION

### eNK cells are highly cytotoxic and may help eradicate HIV

NK cells freshly isolated from PB have limited therapeutic use, but several methods for enhancing NK cells have been described (81). Here, we demonstrated that NK cells from PLWH can be expanded *in vitro* using aAPCs expressing membrane-bound IL-21 (C9.K562-mbIL21). This reproducibly produces CD56^bright^ CD16^+^ eNK cells with high levels of NK cell-activating receptors (48, 55, 56). Although NK cells isolated from PBMCs of PLWH exhibit deficiencies (45, 82–87), our expansion protocol reversed these defects. eNK cells from PLWH expressed the same high levels of activating receptors necessary for cytotoxicity as those from HIV-negative donors. eNK cells also upregulated key cytotoxic markers (granzyme B, perforin, IFNγ, TNFα, Fas ligand, and TRAIL) and were enriched in pathways related to glycolysis, oxidative phosphorylation, and cell cycle proliferation (56, 88). Increased glycolysis and oxidative phosphorylation may enhance cytotoxicity by boosting motility, degranulation, granule regeneration, and production of IFN and ATP (89–92). In addition, eNK cells express FcγRIIIA (CD16A), which mediates ADCC using anti-HIV bNAbs. We demonstrated highly specific targeting of GFP+ HIV-infected cells by eNK cells compared to uninfected cells, along with a significant reduction in HIV reservoirs after latency reversal, as evidenced by decreases in virus release, proviral load, and inducible cell-associated, singly spliced HIV *env* mRNA.

Two Phase I clinical trials (Clinicaltrials.gov NCT03899480 and NCT03346499) evaluated the safety of adoptive transfers in PLWH using eNK cells from HIV-negative donors who are partially HLA haplotype-matched. PBMCs depleted of T and B cells were activated *ex vivo* with IL-2 or N-803, producing a median of 1.1x10^9^ NK cells (1.3 × 10^7^ NK cells/kg) and 3.5 × 10^9^ monocytes. These cells were then infused along with either IL-2 or N-803. These treatments caused no persistent adverse effects and led to moderate reductions in viral RNA+ cells in lymph nodes, but there was no clear impact on HIV DNA+ cells (93). Alternatively, NK cells can be activated and expanded *in vivo* with cytokines without a prior *ex vivo* expansion. A Phase 1 study (NCT02191098) showed that N-803 administered into PLWH on ART resulted in increased activation and higher numbers of CD4+ T cells, CD8+ T cells, and NK cells (94). This treatment correlated with an increase in plasma viral load and acute activation of HIV transcription in memory CD4+ T cells, which was followed by a decline in inducible spliced HIV mRNA for up to 6 months. While these approaches are promising, they may be limited by the low abundance of NK cells in peripheral blood. NK cell number and half-life can be enhanced through expansion with aAPCs expressing membrane-bound IL-21, resulting in several 1000-fold expansion of eNK cells with no signs of senescence or exhaustion that are now being used for cancer therapy (48, 54–56). Clinical studies show eNK cells in the range of 1 × 10^7^ to >4 × 10^8^ NK cells/kg being safely administered (Clinicaltrials.gov NCT02890758) (95–98). Preliminary trials for HIV clearance could therefore use ~1 × 10^8^ NK cells/kg or more without adverse effects, and eNK cell infusions could be safely repeated.

### Targeting HIV reservoirs by eNK cells requires latency reversal

Shock and kill strategies using activated immune effector cells can effectively decrease latently infected HIV cells *in vitro*. For example, IL-15-activated primary NK cells from PLWH kill autologous CD4+ T cells and reduce HIV-infected cell levels *in vitro*, as shown by QVOA (99). HIV-specific CTLs target cells containing HIV provirus after LRA treatment but failed to reduce replication-competent virus as measured by QVOA or murine viral outgrowth (MuVOA) (100). Convertible CAR-T cells reduced inducible proviruses in CD4+ T cells from PLWH after treatment with PMA, ionomycin, and synthetic bNAbs fused to a MIC domain (NKG2D ligand) (101).

In this study, we demonstrated that eNK cells can reduce HIV reservoirs *in vitro* using nucleic acid-based assays. Because defective proviruses in PLWH contaminate conventional DNA-based assays, we independently developed the potentially intact proviral load (PIPL) assay using duplex digital PCR to detect individual proviruses positive for two highly conserved targets separated by several kilobases in the HIV genome. This is similar in design to the intact proviral DNA assay (IPDA) (77, 78), which employs oil-water emulsions of each sample in a duplex digital droplet PCR (ddPCR) format along with highly diluted replicates with cellular probes to correct for DNA shearing. We have found no evidence of significant DNA shearing in the PIPL assay, which is interesting given that our digital PCR platform uses oil-free solutions directly applied to microchips instead of oil-based droplets. The IPDA also uses cellular probes to count cell equivalents, whereas we use DNA concentration and the known mass of the human genome.

Using the PIPL assay, we observed significant reductions in both potentially intact and potentially defective proviruses following treatment with eNK cells. This indicates that all cells expressing HIV-1 are susceptible to targeting and elimination by eNK cells. HIV-1 Nef and Vpu proteins downregulate MHC-I, preventing the ligation of NK cell-inhibitory receptors, while HIV Vpr and Env increase the expression of ligands recognized by NK cell-activating receptors like NKG2D (44) and NKp44 (102). Cells with defective proviruses can still produce HIV antigens, which may lead to immune cell exhaustion. Therefore, reducing potentially defective reservoirs is an integral part of HIV eradication (103) that may be achieved by NK cell therapies.

We also used our HIV RNA-induction assay, the EDITS assay (79), on a broad range of well-suppressed PLWH samples to demonstrate that eNK cells decrease inducible cell-associated HIV mRNAs encoding HIV Env, which are essential for infectivity. Although there was no significant sex-based difference in our limited data set, sex hormones can influence NK cell effector function. Future studies could explore potential differences between premenopausal and postmenopausal women living with HIV (104).

In a shock and kill strategy, LRAs would also be present during immunotherapy. Therefore, we assessed the impact of LRAs on eNK cell-mediated cytotoxicity *in vitro*. HDACi and ingenol were relatively well-tolerated, but bryostatin and prostratin significantly reduced cytotoxicity and are therefore unsuitable for use in combination with eNK activation strategies. SAHA does not impair activated NK cell function at physiological doses, while IL-15 (or the IL-15 superagonist N-803) activates NK cells (53, 99). Our results show that SAHA and IL-15 synergize to promote latency reversal; hence, combining these agents should enhance eNK cell therapy.

Adoptive immunotherapy does not necessarily require that eNK cells be autologous. Early successes with allogeneic NK cells led to the development of more renewable resources that can be used either “off-the-shelf” or on demand. However, the undesirable variability in NK cells derived from PBMCs or cord blood has prompted the use of induced human pluripotent stem cells (iPSC). iPSC-derived NK cells have a more reproducible phenotype because they are derived from extensively characterized clonal master cell lines that are often genetically engineered for further refinement. Several iPSC-derived NK cell products from FATE Therapeutics are now in Phase 1 clinical trials for cancer immunotherapy (105, 106). Successful HIV eradication will also require targeting major HIV reservoirs in lymph nodes, especially follicular T helper cells, and other tissue-specific reservoirs in the gut and brain. Therefore, genetic engineering of eNK cells or iPSC-derived NK cells may enhance HIV eradication by directing therapy to immune-privileged reservoirs.

## MATERIALS AND METHODS

### Human subjects

Leukapheresis packs from HIV-1-negative donors were from AllCells. PBMCs from well-suppressed ART-treated PLWH of UCSF SCOPE cohort and Rustbelt CFAR repository were used. UCSF SCOPE cohort had median age 40 (30–54) years, median nadir CD4 count 211 (76–661) cells/μL, median viral suppression of 37 (19–131) months, and 5–16 years of HIV infection (79). Rustbelt CFAR repository PLWH characteristics were median age 50 (30–61) years, median nadir CD4 count 211.5 (0–531) cells/μL, median CD4 count 887 (492–1,296) cells/μL, and a median duration on ART was 8 (3–21) years (107). Virus release assays used three PLWH from the Pittsburgh AIDS Center for Treatment, selected for suppressive ART regimens for ≥ 24 months with CD4 counts >300 cells/µL within the past 12 months, with no active coinfections with HBV or HCV and no known malignancies.

### HIV-1 molecular clones

We used the following HIV molecular clones: single round HIV-1 proviral clone containing *tat, rev, env, vpu*, and mCD8a-GFP-IRES-*nef* (PHR’ NL4-3-mCD8a-eGFP-IRES-Nef) (80) and full-length replication-competent HIV-1 containing eGFP-IRES-*nef* (pNL4-3-GFP-IRES-Nef (NLgNef) and pNLAD8-GFP-Nef (AD8gNef) (108, 109).

### Cell lines

HIV-transduced Jurkat E6.1 cells (3C9) (110), K562 ATCC CCL-243, C9.K562-mbIL21, and NKF were maintained in RPMI-1640 (Gibco Fisher Scientific) with 10% FBS (Gemini Bio-Products) and normocin (0.1 mg/mL) (InvivoGen). K562-RFP cell line was created by transduction of K562 cells with pRSI9-U6-(sh)-UbiC-TagRFP-2A-Puro vector (Cellecta) and puromycin resistance.

### Acute HIV-1 infection of CD4+ T cells

Memory CD4+ T and total CD4+ T cells were isolated from cryopreserved PBMCs using EasySep Memory CD4+ T cell and EasySep Human CD4+ T cell enrichment kits (StemCell technologies). T cells were activated with PHA-P (5 μg/mL) (Millipore Sigma) or IL-15 (10 ng/mL) (PeproTech) and infected with replication-competent HIV-1 [pAD8gNef (109) or pNLgNef (108)] or transduced with single-round HIV-1 (pHR-CD8a-d2eGFP-IRES-Nef) and Lenti-X Accelerator (TaKaRa).

### Expansion of NK cells

NK cells were activated and expanded as previously described with C9.K562-mbIL21 (48, 55) or NKF cells (56). NK cells were isolated from cryopreserved PBMCs by negative selection using EasySep NK cell enrichment kits (StemCell technologies) or by T cell depletion using human CD3 MicroBeads (Miltenyi Biotec) and cultured in SCGM (CellGenix) supplemented with 10% FBS, normocin, and IL-2 (100–120 IU/mL) with γ-irradiated (100 Gy) aAPC (C9.K562-mbIL21 at a 2:1 ratio or NKF at a 5:1 ratio (aAPC:NK cells)). Irradiated aAPCs were added weekly for 3–4 weeks.

### Assessment of NK cell function

#### Surface marker expression

NK cells were stained with fluorescent antibodies for 20–30 min, washed, resuspended in PBS, and analyzed with a BD LSR Fortessa (BD Biosciences) and WinList 3D 9.0.1 (Verity Software House) software. Anti-human antibodies were from BD Biosciences: CD56 (NCAM16.2), CD3 (HIT3a), NKp30 (p30-15), and NKp44 (p44-8), CD16 (3G8), NKG2D (1D11), NKp46 (9E2), DNAM1 (DX11), 2B4 (2-69), and CD57 (NK-1); from R&D Systems: NKG2A (131411), NKG2C (134591), KIR3DL1 (177407), and KIR2DL1 (143211); and from Miltenyi Biotec KIR2DL2/DL3 (DX27). We used the HIT3a anti-CD3 antibody clone, which stains only surface CD3ε, whereas the UCTH1 clone stains both surface and intracellular CD3ε without permeabilization.

#### NK killing assays by flow cytometry and microscopy

PanToxiLux assay detects killing of K562 and HIV-infected CD4 T cells by fluorogenic cleavage of PS substrate. K562-RFP cells and time-lapse imaging were also used to measure NK cell-mediated cytotoxicity. Further details are in Supplemental Methods. Killing of HIV-GFP-infected CD4+ T cells was detected by loss of GFP and gain of dead cell stain (propidium iodide, PI (Cayman Chemicals). To optimize (HIV)GFP+ cell numbers, acutely infected T cells were mixed with autologous uninfected T cells, and dead cells were removed (Dead Cell Removal Kit, Miltenyi Biotec). NK cells stained with 1 μM CellTrace Violet were mixed with CD4+ T cells acutely infected with replication-competent HIV at 1:1 and 1:10 (NK:target) ratios. For ADCC, antibodies (see Supplemental Methods) were then added. After 24 h, dead cells were stained with PI (5 µg/mL) for 15 min, washed, and fixed with 4% formaldehyde (methanol-free, Polysciences). Loss of GFP indicated HIV(GFP+)-infected CD4+ T cell killing while killing of any cell was stained by PI. Percent killing is percent of GFP+/CTV-negative/PI-negative events from NK cell-treated cultures relative to no NK cells, with all samples in triplicate.

### Single-cell RNA-seq library preparation and data analysis

NK cells were isolated from cryopreserved PBMCs of PLWH. Some cells were expanded with C9.K562-mbIL21 aAPCs for 2 weeks. NK cells were resuspended in 0.04% BSA/PBS and processed for scRNA-seq. Cell viability was assessed by trypan blue staining and Countess II FL (Thermo Fisher), and ~10,000 viable cells per sample were loaded onto a 10× Genomics Chromium Single Cell 3’ Chip (v3.1 chemistry) for gel bead-in-emulsion (GEM) generation. Polyadenylated RNA from each cell was reverse-transcribed into cDNA within individual GEMs, incorporating an Illumina R1 primer sequence, a 10× barcode, and a unique molecular identifier (UMI). After reverse transcription, emulsions were broken and the cDNA was purified with Silane DynaBeads. cDNA was amplified by PCR for 13 cycles, and sequencing libraries were prepared using SPRIselect reagent for size selection. During library construction, Illumina R2 primer sequence, P5 and P7 adapters, and a sample index were added to enable paired-end sequencing. Libraries were sequenced on an Illumina NovaSeq 6000 with 150 cycles. Analysis details are in Supplemental Methods.

### eNK cell killing using EDITS, PIPL assay, and virus release

NK cells were isolated from cryopreserved PBMCs of PLWH. For virus release, NK cells were expanded with irradiated NKF cells. For EDITS and proviral load measurements, NK cells were expanded with C9.K562-mbIL21. CD4+ T cells isolated from autologous donors were treated with LRA for 24 h. When using Dynabeads Human T activator CD3/CD28 (ThermoFisher Scientific) for TCR stimuli, beads were magnetically removed at 24 h. For SAHA (500 nM) and IL-15 (10 ng/mL) or N-803 (1 nM) (Altor Bioscience) combinations, LRAs remained in culture. For virus release assays, cultures of 20–40 million unstimulated or stimulated (LRA-treated) T cells and equal numbers of eNK cells (1:1 ratio of NK:CD4+T cells) were established. For killing assays using EDITS and PIPL assays, cultures of 2–3 million CD4+ T cells and eNK cells (1:1 ratio) were established. All cocultures contained 10 µM raltegravir (Sigma-Aldrich) and 0.4 µM nevirapine (Sigma-Aldrich). For *de novo* virus release, at day 5, a complete media change was performed. At day 7, one-third to one-half of the culture supernatants were collected, centrifuged at 1,000 × *g* for 10 min, flash frozen in dry ice/ethanol, and stored at −80°C. For EDITS and PIPL assays, NK cells were removed prior to analysis (EasySep Human CD56 Positive Selection Kit II, StemCell Technologies). Prior to lysis, CD4+ T cells CD56, CD3, and CD4 levels were analyzed by flow cytometry to confirm removal of NK cells, and the remaining CD4+ T cells (~1–3 million viable cells) were lysed and processed for DNA/RNA analysis.

### Detection of inducible cell-associated HIV-1 mRNA by EDITS

EDITS assays were performed as previously described (79) with modifications shown in Supplemental Methods.

### HIV PIPL assay by duplex digital PCR

Cellular DNAs from memory or total CD4+ T cells of PLWH were isolated using Qiagen DNeasy Blood & Tissue kits but eluted in Qiagen EB Buffer and quantified by Qubit dsDNA HS assay. Proviral loads were measured by the QuantStudio 3D Digital PCR System (Life Technologies). Detailed methods are in Supplemental Methods.

### Measurement of HIV-RNA (virus release)

HIV RNA copies were counted using an Aptima HIV-1 Quan Dx assay on the Panther platform (Hologic). This transcription-mediated amplification method amplifies and detects *pol* and LTR in HIV-1 mRNA. Multiple test values that could not be averaged due to non-detectable or unquantifiable results were assigned half the lower limit of detection (LLoD = 12 cp/mL). Detectable but unquantifiable results were assigned the lower limit of quantitation (LLoQ = 30 cp/mL).

### Statistics

Student’s paired *t* tests were performed using Origin (OriginLab Corporation) or GraphPad Prism. *P* values <0.05 were considered significant. Figure legends indicate numbers of independent experiments or individual donor samples.

## Data Availability

All data for scRNAseqscRNA-seq are available under Gene Expression Omnibus (GEO) accession number GSE312273.

## SUPPLEMENTAL MATERIAL

Fig. S1 mbio.02956-25-s0001.tif Expansion of NK cells from PLWH yields CD56^bright^ eNK cells that express high levels of activating receptors.

Fig. S2 mbio.02956-25-s0002.tif Assays to detect eNK cell-mediated killing of target cells by flow cytometry.

Fig. S3 mbio.02956-25-s0003.tif Differentially expressed genes from curated pathways in eNK cells.

Fig. S4 mbio.02956-25-s0004.tif eNK cells have a different transcriptional profile than non-expanded primary NK cells.

Fig. S5 mbio.02956-25-s0005.tif Volcano plots displaying differentially expressed (DE) genes of eNK cells compared to primary NK cells.

Fig. S6 mbio.02956-25-s0006.tif Specific eNK cell-mediated killing of primary CD4^+^ T cells infected with HIV.

Fig. S7 mbio.02956-25-s0007.tif Enhanced specificity of eNK cells against HIV-infected cells by ADCC using bNAbs.

Fig. S8 mbio.02956-25-s0008.tif Analysis of eNKs and LRAs used for *in vitro* reduction of HIV reservoirs.

Supplemental Methods mbio.02956-25-s0009.docx Detailed methods.

Videos S1 and S2 mbio.02956-25-s0010.mp4 Time lapse of control (S1) and eNK cell killing of activated 3C9 cells.

ASM does not own the copyrights to Supplemental Material that may be linked to, or accessed through, an article. The authors have granted ASM a non-exclusive, world-wide license to publish the Supplemental Material files. Please contact the corresponding author directly for reuse.
