# Supplementary figures and images for: Reduction of the HIV-1 reservoir in T cells from people with HIV-1 on suppressive antiretroviral therapy using expanded natural killer cells
Source: mBio. 2026 Feb 5;17(3):e02956-25. doi: 10.1128/mbio.02956-25 (PMC12977593; doi:10.1128/mbio.02956-25)

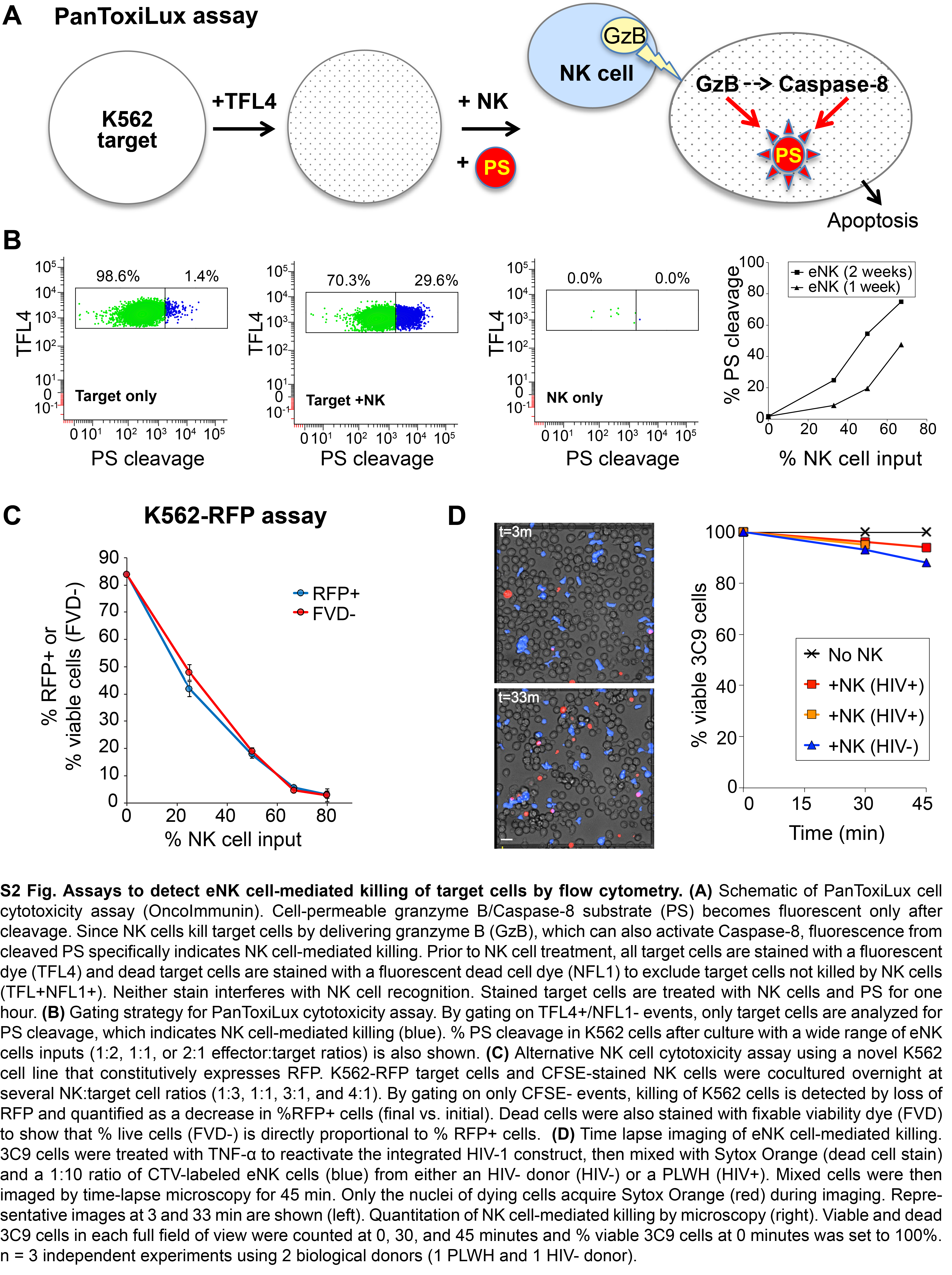

Supplement: Fig. S2 — Assays to detect eNK cell-mediated killing of target cells by flow cytometry. [file mbio.02956-25-s0002.tif]

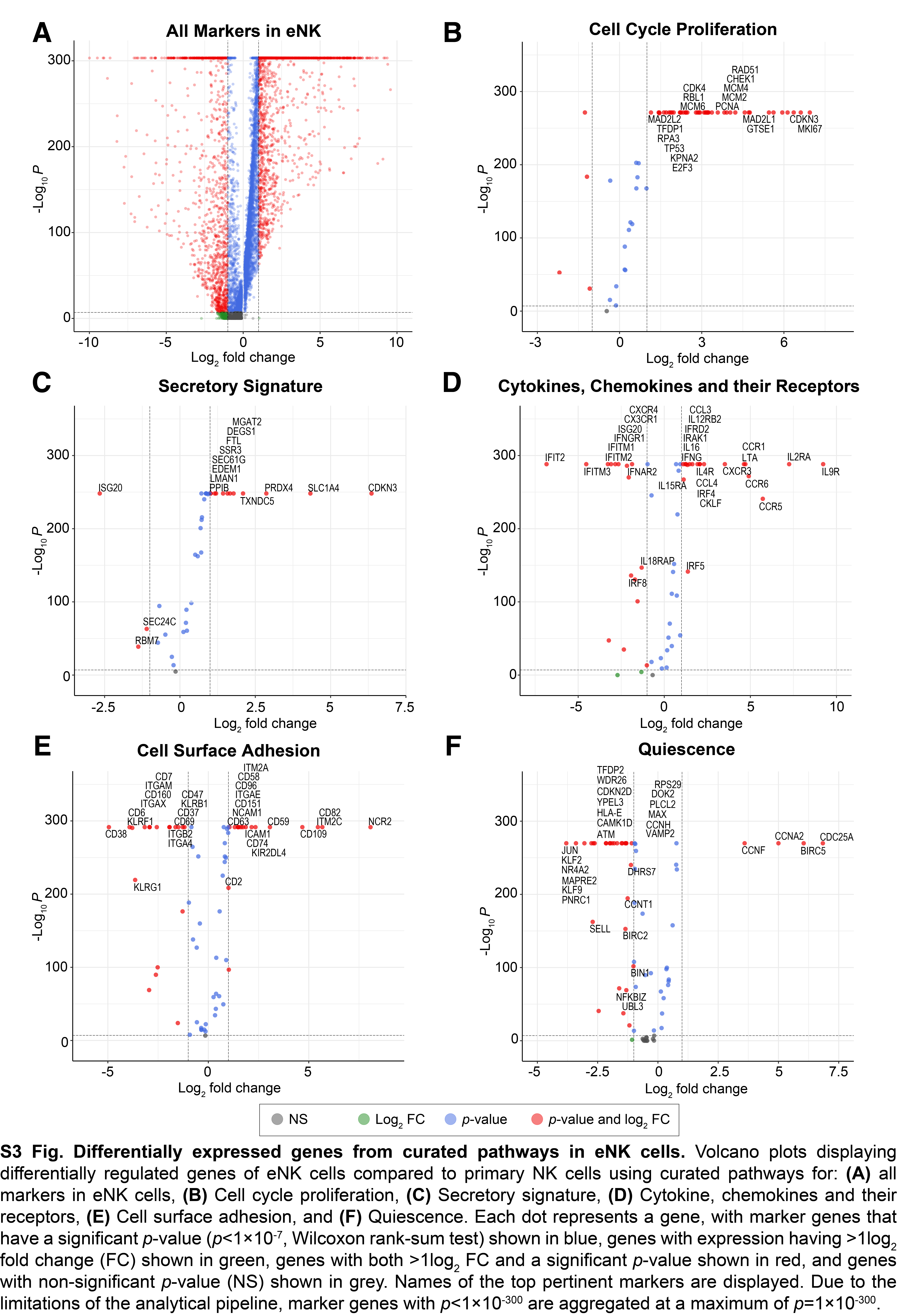

Supplement: Fig. S3 — Differentially expressed genes from curated pathways in eNK cells. [file mbio.02956-25-s0003.tif]

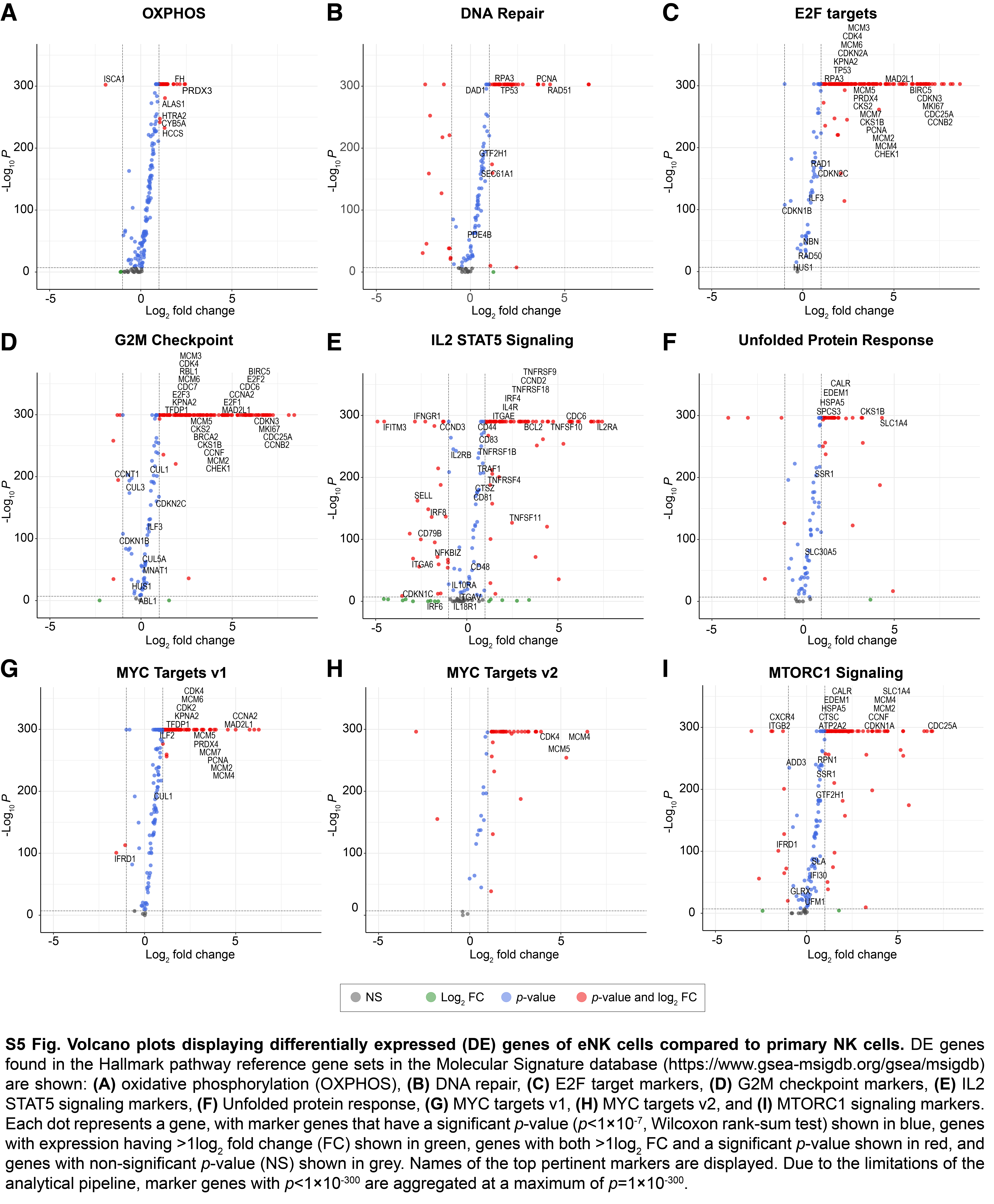

Supplement: Fig. S5 — Volcano plots displaying differentially expressed (DE) genes of eNK cells compared to primary NK cells. [file mbio.02956-25-s0005.tif]

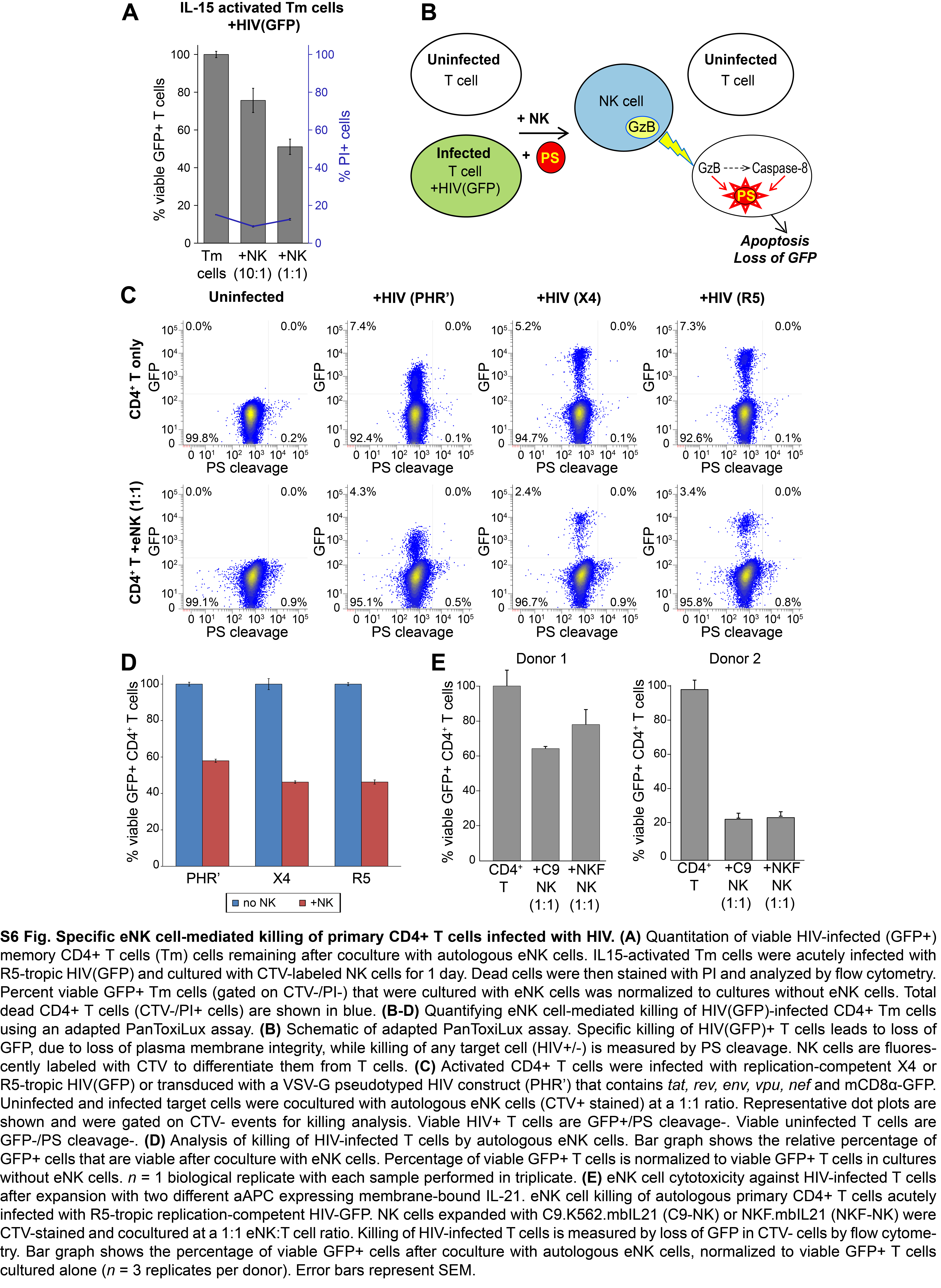

Supplement: Fig. S6 — Specific eNK cell-mediated killing of primary CD4+ T cells infected with HIV. [file mbio.02956-25-s0006.tif]

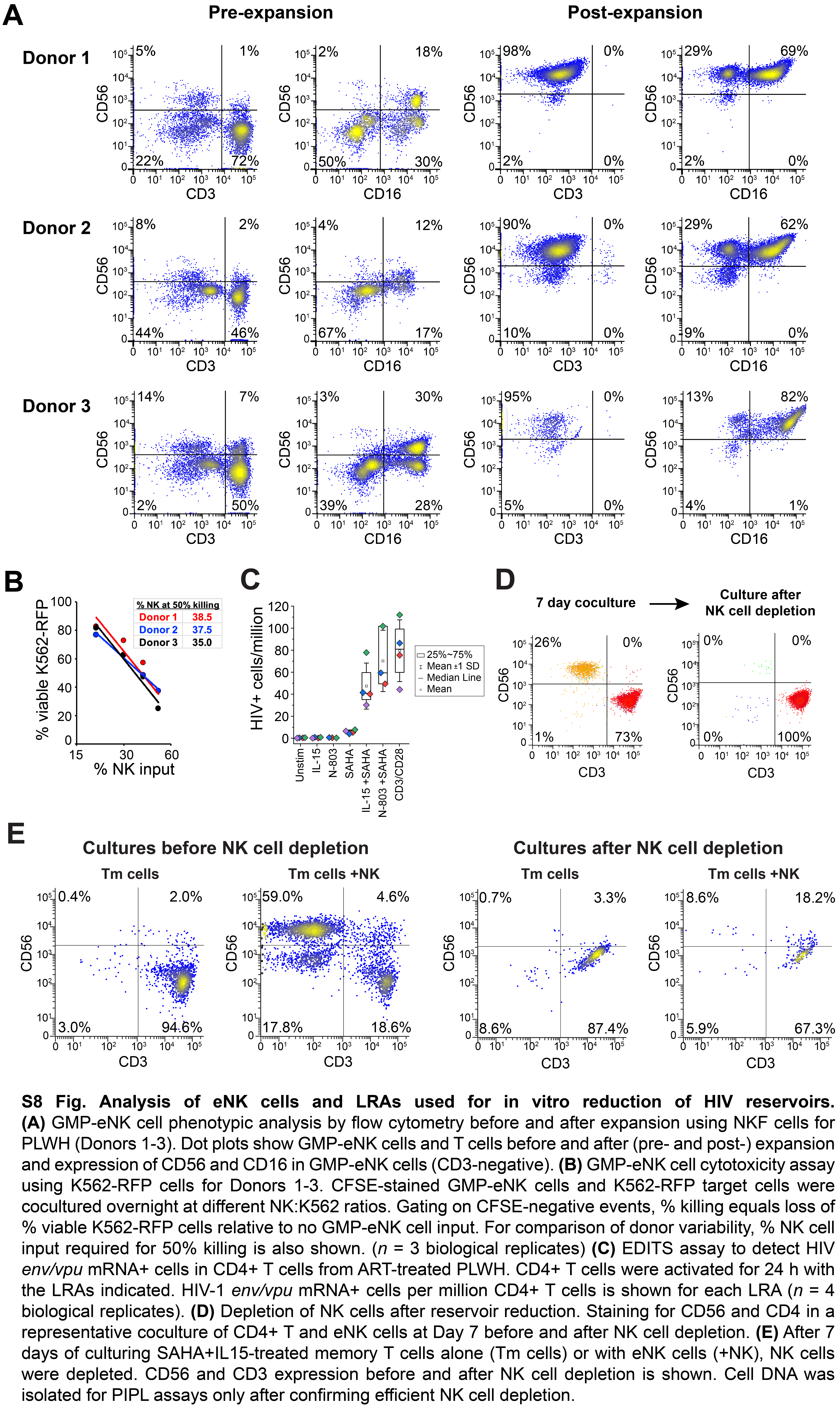

Supplement: Fig. S8 — Analysis of eNKs and LRAs used for in vitro reduction of HIV reservoirs. [file mbio.02956-25-s0008.tif]
