# Supplementary material for: Reduction of the HIV-1 reservoir in T cells from people with HIV-1 on suppressive antiretroviral therapy using expanded natural killer cells
Source: mBio. 2026 Feb 5;17(3):e02956-25. doi: 10.1128/mbio.02956-25 (PMC12977593; doi:10.1128/mbio.02956-25)
Supplement: Supplemental Methods — Detailed methods. [file mbio.02956-25-s0009.docx]

Supplementary Materials and Methods

Assessment of NK cell function

### Flow cytometry NK killing assays

The PanToxiLux (OncoImmunin, Inc.) assay measures killing of target cells by NK cells and was set up following the manufacturer’s protocol. Briefly, target cells (K562 or CD4^+^ T cells) were stained with TFL4 (to discriminate between T cells and NK cells) and NFL1 to differentiate dead cells prior to coculture with NK cells. In some experiments, target cells were not stained with TFL4 but instead NK cells were stained with 1 μM CellTrace Violet (Cell Proliferation dye, ThermoFisher Scientific) following the manufacturer’s protocol, to help differentiate between target cells and effector cells. Cocultures of target cells and eNK cells were established with different eNK cell inputs and incubated for 1 h in the presence of a fluorogenic granzyme B/caspase 8 substrate (PS). The PanToxiLux assay measures target cells that have received a lethal dose of granzyme B from NK cells after coculture, which is detected by PS cleavage.

NK cell cytotoxicity was also measured by their ability to kill K562-RFP target cells. K562-RFP cells were cocultured with different inputs of CellTrace Violet-stained NK cells. To determine the effect of LRAs on NK cell cytotoxicity, NK cells were treated with LRAs [330 nM SAHA (Cayman Chemical), 10 nM romidepsin (Selleck Chemicals), 20 nM panobinostat (Selleck Chemicals), 5 nM bryostatin (Sigma-Aldrich), 1 µM prostratin (Sigma-Aldrich) and 100 nM ingenol (Santa Cruz Biotechnology)] for 24 h prior to staining with CellTrace Violet. Killing of K562-RFP cells leads to loss of RFP and it directly correlates with gain of a dead cell stain (Fixable Viability Dye, eBioscience). Dead cells were stained following the manufacturer’s protocol.

ADCC antibodies

The following reagents were obtained through the NIH HIV Reagent Program, Division of AIDS, NIAID, NIH: anti- HIV-1 gp120 monoclonal antibodies: PGT121 (ARP-12343, contributed by International AIDS Vaccine Initiative)(1), PGT126 (ARP-12344, contributed by International AIDS Vaccine Initiative)(1), PGT128 (ARP-13352, contributed by International AIDS Vaccine Initiative)(1), 10-1074 (ARP-12477, contributed by Dr. Michel Nussenzweig)(2), 2191 (ARP-11682, contributed by Dr. Susan Zolla-Pazner)(3), VRC01 (ARP-12033, contributed by Dr. John Mascola)(4), G54W (NIH45-46 G54W, ARP-12174, contributed by Dr. Pamela Bjorkman)(5), VRC03 (ARP-12032, contributed by Dr. John Mascola)(4), CH106 (ARP-12566, contributed by Drs. Barton F. Haynes and Hua-Xin Liao), 3BNC117 (ARP-12474, contributed by Dr. Michel Nussenzweig)(2), VRC-CH31 (ARP-12565, contributed by Drs. Barton F. Haynes and Hua-Xin Liao), PG9 (ARP-11557, contributed by International AIDS Vaccine Initiative)(6), PG16 (ARP-12150, contributed by International Aids Vaccine Initiative)(6), PGT145 (ARP-12703, contributed by International AIDS Vaccine Initiative)(1), HG107 (ARP-12553, contributed by Duke Human Vaccine Institute, Duke University Medical Center)(7), and CH58 (ARP-12550, contributed by Drs. Barton F. Haynes and Hua-Xin Liao); anti- HIV-1 gp41 monoclonal antibodies: 7B2 (ARP-12556, contributed by Drs. Barton F. Haynes and Hua-Xin Liao), 7B2-AAA (ARP-12557, contributed by Duke Human Vaccine Institute, Duke University Medical Center)(8), 2F5 (ARP-1475, contributed by DAIDS/NIAID)(9), 7H6 (ARP-12295, contributed by Drs. Jinghe Huang, Leo Laub, and Mark Connors), and 10E8 (ARP-12294, contributed by Dr. Mark Connors)(10). PGT121, PGT128, PGT135 and PGT145 (IAVI NAC) were also obtained from IAVI NAC. Anti-human Influenza hemagglutinin monoclonal antibody (CH65, ARP-12555, contributed by Drs. Barton F Haynes and Hua-Xin Liao) was used as a negative control for ADCC and was obtained through the NIH HIV Reagent Program, Division of AIDS, NIAID, NIH.

### NK cell-mediated killing detected by microscopy

Dead cells were removed from target cell cultures and CellTrace Violet-stained NK cells were added at a 1:10 (NK:T cell) ratio. After adding 1 µM Sytox Orange Dead Cell Stain (ThermoFisher Scientific) the cells were loaded into a hemacytometer and imaged with a DeltaVision Core Deconvolution microscope, equipped with a solid-state light source and an environmental conditions of 37° C with humidified air containing 5% CO_2_. Time lapse images were acquired at 30 second intervals for 45 minutes per sample using a CoolSnap HQ camera, 40X lens, and SoftWoRX Pro 6.5.1 software (Applied Precision).

**Single cell RNAseq library preparation and data analysis**

Following NGS, FASTQ files were mapped to a human GRCh38 (GENCODE v44/Ensembl110 annotations) reference set using Cellranger-8.0.0 software (10x Genomics). Seurat package for R was used to generate Seurat objects from the resulted gene count matrices with the following settings: min.cells = 1, min.features = 500 (11, 12). Four Seurat objects were created. Data in the objects was log-normalized, scaled, and variable features and principal components were identified. Significant principal components were determined using ElbowPlot and Jackstraw analyses. This was followed by finding neighbors, clusters and UMAP dimensionality reduction of the first ten significant principal components. Cell cycle stages of the single cells were estimated using the CellCycleScoring function in Seurat. Cell types were determined using SingleR package for R and ENCODE reference dataset (13). Due to heterogeneity of the cell types in the datasets, we manually gated on NK cells using the CellSelector function in Seurat (see R code). The four Seurat objects were merged, followed by a standard object processing that included normalization, scaling, finding variable features, PCA, finding neighbors, finding clusters and UMAP reduction. The UMAP images were generated using the DimPlot function in Seurat. The expression of individual genes was visualized using the FeaturePlot function. Violin plots showing gene expression in expanded versus primary NK cells were built using the VlnPlot function in Seurat.

Differentially expressed (DE) genes in primary vs expanded NK cells were identified using the FindAllMarker function in Seurat that utilized Wilcoxon Rank Sum test. The function was set at log_2_-fold change >0.5 and min.pct=0.1. The function performed searches for both positive and negative gene markers and calculated unadjusted and Bonferroni-adjusted *p*-values. The list of DE genes was then used for pathway enrichment analysis. Two pathway references were used: the Hallmark dataset from Molecular Signature Database (14, 15) and a curated NK cell-specific dataset (16). Enrichment analysis was performed using the *hypeR* package for R (17) with test set to hypergeometric. Dot plots were built in Seurat showing percent of detected DE genes in each pathway reference gene set and negative log_10_ of false discovery ratio (‑log(FDR)). The same DE genes and reference pathway datasets, as well as the *EnhancedVolcano* package for R, were used to generate volcano plots showing log_2_-fold change and *p*-values for the DE gene hits overlapping with the pathway gene sets (https://github.com/kevinblighe/EnhancedVolcano).

eNK cell-mediated killing assay against autologous CD4^+^ T cells from PLWH for HIV reservoir detection by EDITS, proviral load measurement and virus release

### Detection of inducible singly spliced HIV-1 *env/vpu* mRNA by EDITS

For RNA isolation, cells were lysed at 55° C for 10 min in 75 µl of lysis buffer [0.1 M Tris pH 8.0, 0.2 M NaCl, 5 mM EDTA, 1% SDS, and 0.2 mg/ml Proteinase K (ThermoScientific)] with shaking at 1500 rpm. After lysis, 25 µl of 100% isopropanol and 180 µl of Cytiva Sera-Mag SpeedBeads Carboxylate-Modified Magnetic Particles (Fisher Scientific) suspended in 2.5 M NaCl, 1 mM trisodium citrate, 20% PEG 8000, 0.05% Tween-20, pH 6.4 were incubated with RNA for 5 minutes and immobilized with a magnet. RNA-bound beads were washed twice with Washing Buffer I (0.5% SDS, 80% EtOH, 1 mM trisodium citrate, pH 6.4) and once with Washing Buffer II (80% EtOH, 1 mM trisodium citrate, pH 6.4). RNA-bound beads were then DNase treated (10 mM Tris-HCl [pH 8], 2.5 mM MgCl2, 0.5 mM CaCl2, and 1 Unit RNase-free DNase) for 15 min at 37° C. A solution of 20% PEG 8000 and 2.5 mM NaCl was added to the DNase-treated RNA-bound beads. Beads were then washed twice with Washing Buffer II and dried. RNA was eluted in 40 µl of 10 mM Tris pH 8.0. One quarter of the total RNA sample was used as template for OneStep RT-PCR (ABM) using the following primers: EDITS Fwd 5’-GTTGTGTGACTCTGGTAACTAG-3’ and EDITS Rvs 5’ CTGAAGATCTCGGACCCATTGT-3’ and cycling conditions: 42° C for 30 min, 94° C for 3 min, 35 cycles of (94° C for 30 s, 55° C for 30 s, 72° C for 60 s), and 72° C for 5 min. A nested PCR was then performed using 4% of OneStep RT-PCR product mixed with BestTaq 2x Master Mix and barcoded primers containing linkers compatible with Ion Torrent Sequencing and the following HIV-1-specific sequences: EDITS nFwd 5’-TAGTCAGTGTGGAAAATCTCTA-3’ and EDITS nRvs 5’-CATAATAGACTGTGACCCACAA-3’. Nested PCRs were amplified with the following conditions: 94° C for 3 min, 5 cycles of (94° C for 10 s, 60° C for 30 sec, 72° C for 10 sec), 30 cycles of (94° C for 10 s, 72° C for 15 s), and 72° C for 3 min. Nested PCR samples were then pooled, purified by agarose gel electrophoresis, quantified with Qubit HS DNA Assay and analyzed by next generation sequencing using the Ion Chef System and Ion S5 System (ThermoFisher Scientific) following the manufacturer’s protocol. Sequences were then mapped to an HIV genome reference and quantified using Geneious Prime software (Geneious).

### HIV Potentially Intact Proviral Load (PIPL) assay by duplex digital PCR

**Duplex digital PCR.** 300-700 ng of cellular DNA per reaction was mixed with QuantStudio™ 3D Digital PCR Master Mix v2 and the following: 300 nM *gag* forward primer 5’-AGCCCAGAAGTAATACCCATGTTT*-*3’ (HXB2 nt 1282-1305), 300 nM *gag* reverse primer 5’-CCCCCCACTGTGTTTAGCATG*-*3’ (HXB2 nt 1347-1367), 300 nM *env* forward primer 5’-TGGAAAAATGACATGGTAGAACAGA-3’ (HXB2 nt 6510-6534), 300 nM env reverse primer 5’-TTTACACATGGCTTTAGGCTTTGA-3’ (HXB2 nt 6563-6586), 300 nM TaqMan® *gag* probe 5’-(6-FAM)-CAGCATTATCAGAAGGAGCC-(MGBNFQ)-3’ (HXB2 nt 1307-1326), and 300 nM TaqMan® *env* probe *5’*(VIC)-CATGAGGATATAATCAGTTTATGG-(MGBNFQ)-3’ (HXB2 nt 6537-6560). PCR samples were loaded in 2-3 replicates onto QuantStudio® 3D Digital PCR 20K Chips v2 using a QuantStudio® 3D Digital PCR Chip Loader. Sealed chips were cycled using a Dual Flat Block GeneAmp™ PCR System 9700 (Applied Biosystems®) at 96°C for 10 minutes, followed by 40 cycles of 60°C for 2 min and 98°C for 30 sec, with a final extension at 60°C for 2 min. Fluorescence was measured with a QuantStudio 3D Digital PCR Instrument.

**Chip Analysis.** Fluorescent wells on each chip were counted with Analysis Suite dPCR Cloud Software (ThermoFisher Connect™ Platform). Although the software provide copies/µl in each channel (FAM or VIC), the “FAM” counts/µl included FAM+VIC+ counts, “VIC” counts/µl included FAM+VIC+ counts, and no separate FAM+VIC+ count/µl was given. Since these counts are not useful for this assay, we developed algorithms to calculate the desired copies/µl of single-positive (FAM only, VIC only) proviruses that were considered potentially defective, and double-positive proviruses (FAMVIC) that were considered potentially intact. The software provided lists of “Calls”, which are numbers of wells with a signal that fell in one of the four possible categories: FAM (*gag+* only), VIC (*env+* only), FAMVIC (*gag+env+*), or “NoAmp” (gag-negative and env-negative), with HIV-negative DNA samples being used to define the thresholds. To calculate copies/µl we needed to find the actual loaded sample volume, so we used the total number of calls per chip. A passive ROX dye indicated wells containing PCR mix, so that calls are only counted in ROX^positive^ wells. Since 15 µl of PCR was spread onto each chip containing 20,000 wells, we determined that each well contains at least 0.75 nl. We estimated the actual loaded sample volume was the total number of calls x 0.755 nl/well, and total calls relative to 20,000 (maximum) was the loading efficiency.

**Correction for undercounting.** “Calls” determined by the software show detection of at least one target copy in any well, but the probability that multiple targets are present in those wells increases as the target concentration increases, due to Poisson distribution. If calls were directly converted to copy number, wells with >1 actual copy would be underestimated. Correction for copies **not** counted by calls was determined for each channel by a modified multiplicity of infection equation, substituting wells on each chip for cells and target DNA copies for infectious units. Thus, the fraction of ROX^positive^ wells containing a given number (n) of targets is [P(n)] by the following:

P(n) = e^-m^m^n^/n!

where m = (target copies loaded per chip) / (total calls per chip). The fractions of wells containing multiple copies (n>2) were added back to the call numbers (FAM, VIC, or FAMVIC) to determine copy numbers, which were then divided by the calculated loaded sample volume to find copies/µl. Data from multiple chips were combined to improve confidence. Following the manufacturer’s suggested protocol, the number of human cells represented in each PCR is based on the concentration of cell DNA and mass of the human diploid genome (6.6x10^-12^*g* DNA/cell). Thus, copies per million cells is [ (copies/µl) / (µg DNA/µl) ] x (6.6 µg DNA / million cells).

1. Walker LM, Huber M, Doores KJ, Falkowska E, Pejchal R, Julien JP, Wang SK, Ramos A, Chan-Hui PY, Moyle M, Mitcham JL, Hammond PW, Olsen OA, Phung P, Fling S, Wong CH, Phogat S, Wrin T, Simek MD, Protocol GPI, Koff WC, Wilson IA, Burton DR, Poignard P. 2011. Broad neutralization coverage of HIV by multiple highly potent antibodies. Nature 477:466-70.

2. Shingai M, Nishimura Y, Klein F, Mouquet H, Donau OK, Plishka R, Buckler-White A, Seaman M, Piatak M, Jr., Lifson JD, Dimitrov DS, Nussenzweig MC, Martin MA. 2013. Antibody-mediated immunotherapy of macaques chronically infected with SHIV suppresses viraemia. Nature 503:277-80.

3. Gorny MK, Williams C, Volsky B, Revesz K, Cohen S, Polonis VR, Honnen WJ, Kayman SC, Krachmarov C, Pinter A, Zolla-Pazner S. 2002. Human monoclonal antibodies specific for conformation-sensitive epitopes of V3 neutralize human immunodeficiency virus type 1 primary isolates from various clades. J Virol 76:9035-45.

4. Wu X, Yang ZY, Li Y, Hogerkorp CM, Schief WR, Seaman MS, Zhou T, Schmidt SD, Wu L, Xu L, Longo NS, McKee K, O'Dell S, Louder MK, Wycuff DL, Feng Y, Nason M, Doria-Rose N, Connors M, Kwong PD, Roederer M, Wyatt RT, Nabel GJ, Mascola JR. 2010. Rational design of envelope identifies broadly neutralizing human monoclonal antibodies to HIV-1. Science 329:856-61.

5. Diskin R, Scheid JF, Marcovecchio PM, West AP, Jr., Klein F, Gao H, Gnanapragasam PN, Abadir A, Seaman MS, Nussenzweig MC, Bjorkman PJ. 2011. Increasing the potency and breadth of an HIV antibody by using structure-based rational design. Science 334:1289-93.

6. Walker LM, Phogat SK, Chan-Hui PY, Wagner D, Phung P, Goss JL, Wrin T, Simek MD, Fling S, Mitcham JL, Lehrman JK, Priddy FH, Olsen OA, Frey SM, Hammond PW, Protocol GPI, Kaminsky S, Zamb T, Moyle M, Koff WC, Poignard P, Burton DR. 2009. Broad and potent neutralizing antibodies from an African donor reveal a new HIV-1 vaccine target. Science 326:285-9.

7. Bonsignori M, Pollara J, Moody MA, Alpert MD, Chen X, Hwang KK, Gilbert PB, Huang Y, Gurley TC, Kozink DM, Marshall DJ, Whitesides JF, Tsao CY, Kaewkungwal J, Nitayaphan S, Pitisuttithum P, Rerks-Ngarm S, Kim JH, Michael NL, Tomaras GD, Montefiori DC, Lewis GK, DeVico A, Evans DT, Ferrari G, Liao HX, Haynes BF. 2012. Antibody-dependent cellular cytotoxicity-mediating antibodies from an HIV-1 vaccine efficacy trial target multiple epitopes and preferentially use the VH1 gene family. J Virol 86:11521-32.

8. Zhang W, Godillot AP, Wyatt R, Sodroski J, Chaiken I. 2001. Antibody 17b binding at the coreceptor site weakens the kinetics of the interaction of envelope glycoprotein gp120 with CD4. Biochemistry 40:1662-70.

9. Buchacher A, Predl R, Strutzenberger K, Steinfellner W, Trkola A, Purtscher M, Gruber G, Tauer C, Steindl F, Jungbauer A, et al. 1994. Generation of human monoclonal antibodies against HIV-1 proteins; electrofusion and Epstein-Barr virus transformation for peripheral blood lymphocyte immortalization. AIDS Res Hum Retroviruses 10:359-69.

10. Huang J, Ofek G, Laub L, Louder MK, Doria-Rose NA, Longo NS, Imamichi H, Bailer RT, Chakrabarti B, Sharma SK, Alam SM, Wang T, Yang Y, Zhang B, Migueles SA, Wyatt R, Haynes BF, Kwong PD, Mascola JR, Connors M. 2012. Broad and potent neutralization of HIV-1 by a gp41-specific human antibody. Nature 491:406-12.

11. Butler A, Hoffman P, Smibert P, Papalexi E, Satija R. 2018. Integrating single-cell transcriptomic data across different conditions, technologies, and species. Nat Biotechnol 36:411-420.

12. Satija R, Farrell JA, Gennert D, Schier AF, Regev A. 2015. Spatial reconstruction of single-cell gene expression data. Nat Biotechnol 33:495-502.

13. Aran D, Looney AP, Liu L, Wu E, Fong V, Hsu A, Chak S, Naikawadi RP, Wolters PJ, Abate AR, Butte AJ, Bhattacharya M. 2019. Reference-based analysis of lung single-cell sequencing reveals a transitional profibrotic macrophage. Nat Immunol 20:163-172.

14. Liberzon A, Birger C, Thorvaldsdottir H, Ghandi M, Mesirov JP, Tamayo P. 2015. The Molecular Signatures Database (MSigDB) hallmark gene set collection. Cell Syst 1:417-425.

15. Subramanian A, Tamayo P, Mootha VK, Mukherjee S, Ebert BL, Gillette MA, Paulovich A, Pomeroy SL, Golub TR, Lander ES, Mesirov JP. 2005. Gene set enrichment analysis: a knowledge-based approach for interpreting genome-wide expression profiles. Proc Natl Acad Sci U S A 102:15545-50.

16. Dybkaer K, Iqbal J, Zhou G, Geng H, Xiao L, Schmitz A, d'Amore F, Chan WC. 2007. Genome wide transcriptional analysis of resting and IL2 activated human natural killer cells: gene expression signatures indicative of novel molecular signaling pathways. BMC Genomics 8:230.

17. Federico A, Monti S. 2020. hypeR: an R package for geneset enrichment workflows. Bioinformatics 36:1307-1308.
